# Efficacy, acceptability, and safety of antidepressants for low back pain: a systematic review and meta-analysis

**DOI:** 10.1186/s13643-021-01599-4

**Published:** 2021-02-24

**Authors:** Michael C. Ferraro, Matthew K. Bagg, Michael A. Wewege, Aidan G. Cashin, Hayley B. Leake, Rodrigo R. N. Rizzo, Matthew D. Jones, Sylvia M. Gustin, Richard Day, Colleen K. Loo, James H. McAuley

**Affiliations:** 1 Centre for Pain IMPACT, Neuroscience Research Australia, Sydney, NSW Australia; 2 School of Health Sciences, University of New South Wales, Sydney, Australia; 3 Prince of Wales Clinical School, University of New South Wales, Sydney, Australia; 4 New College Village, University of New South Wales, Sydney, Australia; 5 IIMPACT in Health, University of South Australia, Adelaide, Australia; 6 School of Psychology, University of New South Wales, Sydney, Australia; 7 Clinical Pharmacology & Toxicology, St. Vincent’s Hospital, Sydney, Australia; 8 St. Vincent’s Clinical School, Faculty of Medicine, University of New South Wales, Sydney, Australia; 9 School of Psychiatry, University of New South Wales, Sydney, Australia; 10 Black Dog Institute, Sydney, Australia

**Keywords:** Low back pain, Antidepressants, Analgesics, Drug therapy, Review, Meta-analysis

## Abstract

**Background:**

Antidepressant medicines are used to manage symptoms of low back pain. The efficacy, acceptability, and safety of antidepressant medicines for low back pain (LBP) are not clear. We aimed to evaluate the efficacy, acceptability, and safety of antidepressant medicines for LBP.

**Methods:**

We searched CENTRAL, MEDLINE, Embase, CINAHL, ClinicalTrials.gov, the EU Clinical Trials Register, and the WHO International Clinical Trial Registry Platform from inception to May 2020. We included published and trial registry reports of RCTs that allocated adult participants with LBP to receive an antidepressant medicine or a placebo medicine. Pairs of authors independently extracted data in duplicate. We extracted participant characteristics, study sample size, outcome values, and measures of variance for each outcome. We data using random-effects meta-analysis models and calculated estimates of effects and heterogeneity for each outcome. We formed judgments of confidence in the evidence in accordance with GRADE. We report our findings in accordance with the PRISMA statement. We prespecified all outcomes in a prospectively registered protocol. The primary outcomes were pain intensity and acceptability. We measured pain intensity at end-of-treatment on a 0–100 point scale and considered 10 points the minimal clinically important difference. We defined acceptability as the odds of stopping treatment for any reason.

**Results:**

We included 23 RCTs in this review. Data were available for pain in 17 trials and acceptability in 14 trials. Treatment with antidepressants decreased pain intensity by 4.33  points (95% CI − 6.15 to − 2.50) on a 0–100 scale, compared to placebo. Treatment with antidepressants increased the odds of stopping treatment for any reason (OR 1.27 [95% CI 1.03 to 1.56]), compared to placebo.

**Conclusions:**

Treatment of LBP with antidepressants is associated with small reductions in pain intensity and increased odds of stopping treatment for any reason, compared to placebo. The effect on pain is not clinically important. The effect on acceptability warrants consideration. These findings provide Level I evidence to guide clinicians in their use of antidepressants to treat LBP.

**Trial registration:**

We prospectively registered the protocol for this systematic review on PROSPERO (CRD42020149275).

**Supplementary Information:**

The online version contains supplementary material available at 10.1186/s13643-021-01599-4.

## Background

Low back pain (LBP) is the leading cause of disability worldwide [1]. The most common interventions for LBP are medicines that aim to reduce symptoms [2–7]. Clinical guidelines for LBP recommend that medicines should be prescribed for those who fail to respond to non-pharmacological interventions [8–11] and restricted to short-term use due to the potential for adverse effects and abuse [11]. Common medicines prescribed for LBP include non-steroidalanti-inflammatories (NSAIDs), opioids, muscle relaxants, and antidepressants [3, 12–14].

Antidepressants are a broad group of medicines classified according to their presumed action [15]. The mechanism of their analgesic effects is not well understood [16, 17]. Antidepressants are prescribed for LBP to provide pain relief, improve sleep, or reduce co-morbid depressive symptoms [18]. There is evidence that prescription rates of antidepressants to manage LBP are increasing [14, 19].

Evidence to support the efficacy and safety of antidepressants for LBP is unclear. Findings from systematic reviews are inconsistent [20–23]. The most recent review found inconclusive evidence for the effect of antidepressant medicines on pain intensity, disability or depression [23], and inadequate evidence to evaluate the acceptability and safety of antidepressants for LBP. The most recently published clinical guidelines for LBP provide conflicting advice on the use of antidepressants for LBP. The American College of Physicians guideline endorses duloxetine for chronic LBP [11] whereas the National Institute for Health and Care Excellence (UK) guideline advises against the use of any antidepressant for LBP [9].

The aim of this systematic review was to evaluate the efficacy, acceptability, and safety of antidepressant medicines compared to placebo for LBP, using data from published and trial registry reports.

## Methods

We prospectively registered the protocol [24] for this systematic review on PROSPERO (CRD42020149275) and report our findings according to the Preferred Reporting Items for Systematic Reviews and Meta-Analyses (PRISMA) guideline [25] (Checklist S1 in Additional file 1).

### Primary outcomes

The primary outcomes were pain intensity and acceptability. Pain intensity was measured at the follow-up assessment closest to the end of treatment. Acceptability, defined as overall acceptability of the medicine, was measured using all-cause discontinuation during treatment [15, 26].

### Secondary outcomes

The secondary outcomes included low back-specific function, symptoms of depression, safety, harm, and tolerability. Low back-specific function and symptoms of depression were measured at the follow-up assessment closest to the end of treatment. Safety and harm, defined as the incidence of adverse effects and serious adverse effects [27], were measured by reports of adverse effects and serious adverse effects during treatment. Tolerability was defined as the tolerability of adverse effects sustained during treatment, measured by reports of discontinued treatment due to adverse effects.

### Data sources

We used comprehensive search strategies to search electronic databases and clinical trial registries for records of randomized clinical trials of antidepressant medicines in LBP (Appendix S1 in Additional file 2) [28, 29]. We piloted the strategies using records of trials included in a previous systematic review [23]. We searched the Cochrane Back and Neck Group’s Trials Register and the Cochrane Central Register of Controlled Trials (CENTRAL) (Cochrane Library), MEDLINE, Embase (Ovid), and CINAHL (EBSCO) databases from inception to May 15, 2020. We searched ClinicalTrials.gov (ClinicalTrials.gov), the EU Clinical Trials Register (www.clinicaltrialsregister.eu), and the WHO International Clinical Trial Registry Platform (apps.who.int/trialsearch/Default.aspx) from inception to May 15, 2020. We included records written in English, Italian, Spanish, Portuguese, German, and French.

We included published and trial registry reports of randomized controlled trials (RCTs) that allocated adult participants with LBP to receive (i) a systemically administered dose of an antidepressant medicine or (ii) a sham (placebo) medicine, (iii) continuation of usual care, (iv) a waiting list, or (v) no-treatment. LBP was defined as pain of any duration between the 12th rib and buttock crease, with or without associated leg pain [30]. Trials that only included participants with symptoms of nerve root compromise (sciatica) [31] or LBP due to specific medical conditions (e.g., spinal fracture, inflammatory disease, aortic dissection, malignancy, or infection) were excluded. We included trials of mixed samples (e.g., non-specific LBP and LBP with sciatica, or non-specific LBP and large joint osteoarthritis) if separate data for the non-specific LBP sample were available. We included trials that tested the efficacy of selective serotonin reuptake inhibitors (SSRIs), serotonin and norepinephrine reuptake inhibitors (SNRIs), tricyclic antidepressants (TCAs), tetracyclic antidepressants (TeCA), heterocyclic antidepressants (HCAs), monoamine oxidase inhibitors (MAOIs), or atypical antidepressants, provided they were listed on the WHO ATC [32] and licensed in at least one of the following jurisdictions: USA (FDA) [33], Australia (TGA) [34], UK (MHRA) [35], or Europe (EMA) [36].

We screened records for inclusion in two stages. Pairs of authors from a team of six (MCF, MAW, AGC, MDJ, HBL, RRNR) independently screened record titles and abstracts in duplicate. The full texts of potentially eligible records were retrieved and independently screened again (MCF, MAW) to confirm inclusion. Disagreements were resolved through discussion or recourse to a third author (MKB or JHM).

We linked records to identify unique studies using a hierarchy. Records that were published and reported the results of a trial were classified as primary records, followed by other published records of a trial (e.g., secondary analyses), conference abstracts, and lastly, trial registry records. We classified the trial registry record as the primary record if there was no evidence of publication.

### Data extraction and risk of bias assessment

Pairs of authors (MCF, MAW, AGC, HBL, RRNR, and MDJ) independently extracted data using standardized, piloted, data extraction forms and assessed study-level risk of bias using the Cochrane “Risk of bias” tool (version 5.1.0) [37] and published recommendations [38, 39]. Outcomes were rated as low overall risk when three or fewer domains are rated “unclear” risk, and no domains were rated “high”; moderate risk if a single domain was rated as “high” risk, but four or more were rated as “unclear” and high overall risk in all other instances. We resolved conflicts by consensus or, where necessary, through arbitration with a third author (MKB, JHM). We extracted, for each trial, the following: participant age, sex, duration of symptoms, and sample size; outcome value and measure of variance for pain intensity, function, and symptoms of depression; number of adverse and serious adverse effects; and the number of participants that discontinued treatment for any reason or due to adverse effects. We used an established hierarchy to preference data from continuous measures of pain, function, and symptoms of depression and converted all outcome data to a 0–100-point scale [24]. We used recommended methods [40, 41] to calculate standard deviations when these were not available.

### Effect measures and interpretation

We used the difference in means and accompanying 95% confidence intervals for analyses of effects of antidepressant medicines on continuous outcomes (pain, function, symptoms of depression). We followed recommended guidance for trials with multiple arms by dividing the control group sample size by the number of arms in the study (Cochrane Handbook, Version 6) [42]. For cross-over trials where we were unable to obtain the first phase outcome data from the study authors, we included the overall effect (reflecting both phases) adjusted to correct for the correlation between the two phases [41]. The minimal clinically important difference in means is established as 10 points on a common 0–100-point scale for both pain and function [42]. We used the odds ratio and accompanying 95% confidence intervals for analyses of effects of antidepressant medicines on binary outcomes (acceptability, safety, harm, tolerability).

### Data synthesis

#### Main analysis

We synthesized the data for each outcome using frequentist random-effectsmeta-analysis models. We fit the models using Restricted Maximum Likelihood (REML) in the R (version 3.6.2) package metafor (version 2.4-0) [43, 44]. We calculated the Q statistic to estimate heterogeneity, the estimate of between-study variance (*τ*^2^), and the proportion of this variance not due to sampling error (*I*^2^). We calculated the 95% prediction interval for the pooled effect and displayed this on the forest plot alongside the pooled effect estimate and 95% confidence interval.

#### Investigation of heterogeneity

We specified symptom duration, medicine type, and dose as covariates for investigation of important heterogeneity in the main analyses. Symptom duration had three levels: 0–6 weeks, 6–12 weeks, and > 12 weeks. Medicine type had seven levels: atypical, HCA, MAOI, SSRI, SNRI, TCA, TeCA. We included an additional level of medicine dose, compared to the protocol: standard dose range (SDR), less than SDR, and above SDR according to the Prescriber’s Digital Reference [45]. We conducted subgroup analyses, using the covariate levels as strata.

#### Sensitivity analyses

We tested the effect of the definition of non-specific LBP and of imputing missing measures of variance by repeating the main analyses with and without the relevant studies.

### Influence of a hypothetical RCT

We constructed extended funnel plots using Stata (version 14.2) [46] to simulate the influence of hypothetical parameters of a future RCT on the pooled effect estimate for pain intensity [47, 48]. The extended funnel plot augments a funnel plot with overlays to provide an illustration of the impact of a new trial on a given meta-analysis [48]. We used 10 points on a 0–100 pain intensity scale as the threshold for the smallest worthwhile effect. We did not perform this analysis for acceptability as there is no known smallest worthwhile effect for this outcome.

### Confidence in cumulative evidence

Two authors (MCF, MAW) used the Grading of Recommendations Assessment Development and Evaluation (GRADE) [49] framework to develop judgements of high, moderate, low, or very low confidence in the evidence for each outcome. We assessed the domains of study limitations, inconsistency, imprecision, and publication bias, using planned criteria [24]. Publication bias was evaluated using visual assessment of funnel plot symmetry, and Egger’s tests where 10 or more studies were available for an outcome [50].

## Results

### Search results

**Fig. 1:**
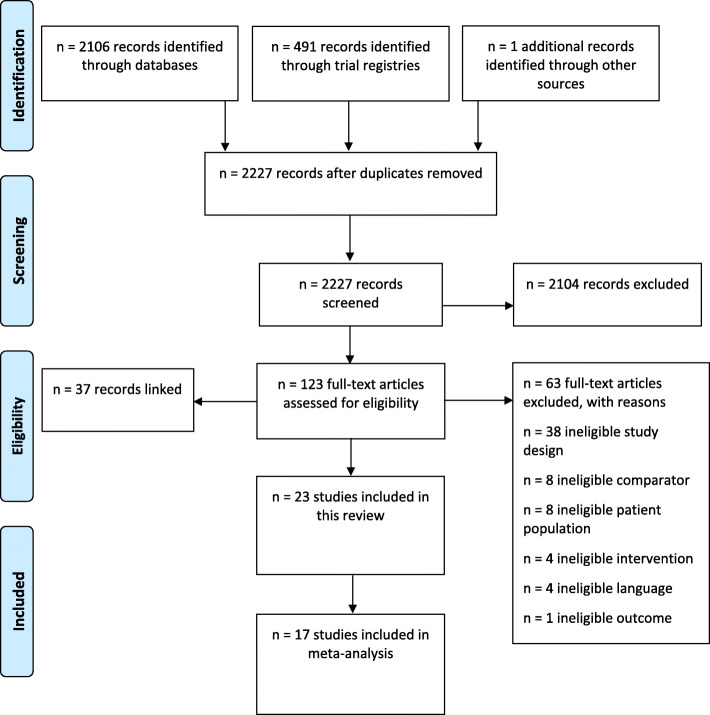
PRISMA flow diagram of the record selection process

The search identified 2598 records. We removed 371 duplicates and screened the titles and abstracts of 2227 records for inclusion. We excluded 2104 records and retrieved the full-texts of 123 potentially eligible records (Fig. 1). We excluded 63 records and included 60 records that comprised 23 unique trials [51–69] (Table 1).

**Table 1:** Characteristics of included studies

Study	Patient sample	Setting	Number of trial arms	Intervention, number assigned	Comparator, number assigned	Duration of treatment	Outcome measures applicable to this review
Label, citation	Study level data unless reported otherwise; mean (SD)			(mg/day unless indicated)	(mg/day unless indicated)		
Alcoff et al. [51]	50 participants with subacute and chronic LBP; mean age imipramine group 29.2 years, placebo group 33.8 years^b^; *n* = 24 (48%) female	USA; 2 sites	2	Oral imipramine 75 for 3 days, 150 thereafter, *n* = 28	Placebo, *n* = 22	8 weeks	SBPQ, BDI
Atkinson et al. [52]	121 participants with chronic LBP; mean age 46.4 (10.2) years; *n* = 47 (38.8%) female	USA	7	Oral desipramine target concentrations of 50 ng/mL *n* = 17, or 110 ng/mL *n* = 17, or 150 ng/mL *n* = 18, or fluoxetine target concentrations of 50 ng/mL *n* = 14, or 100 ng/mL *n* = 14, or 150 ng/mL *n* = 15	Active placebo (benztropine mesylate) *n* = 26	12 weeks	DDS, BDI, RMDQ
Atkinson et al. [62]	103 participants with chronic LBP; mean age 49.2 (9.4) years; *n* = 38 (37%) female	USA	3	Oral maprotiline 150, *n* = 33, or paroxetine 30, *n* = 34	Active placebo (diphenhydramine) 37.5, *n* = 36	8 weeks	DDS, BDI
Atkinson et al. [63]	78 participants with chronic LBP; mean age nortriptyline group 45.79 (10.59) years, placebo group 47.13 (10.65) years; *n* = 0 (0%) female	USA	2	Oral nortriptyline 25 for 3 days, 50 for 4 days, 75 for 3 days, 100 for 4 days to reach target concentration of 50–150 ng/ml, *n* = 38	Placebo, *n* = 40	8 weeks	DDS, BDI
Dickens et al. [64]	92 participants with chronic LBP. Mean age 45 years^b^; *n* = 50 (54%) female	UK	2	Oral paroxetine 20, *n* = 44	Placebo, *n* = 48	8 weeks	100 mm VAS, MADRS
Goodkin et al. [65]	42 participants with chronic LBP; mean age 53.6 (12.9) years; *n* = 16 (38%) female	USA	2	Oral trazodone 50, increasing to 600, *n* = 22	Placebo, *n* = 22	6 weeks	100 mm VAS, BDI
Gould et al. [66]	142 participants with chronic LBP; mean age 55.8 (11.7) years; *n* = 15 (11 %) female	USA	4	Oral desipramine hydrochloride to reach target concentration of 5–60 ng/ml, *n* = 37, or desipramine hydrochloride to reach target concentration of 5–60 ng/ml and cognitive behavioral therapy, *n* = 37	Active placebo (benztropine mesylate) 0.125 and cognitive behavioral therapy, *n* = 33, or active placebo (benztropine mesylate) 0.125, *n* = 32	12 weeks	DDS, RMDQ
Jenkins et al. [67]	59 participants with acute and chronic LBP; mean age imipramine group 26 years, placebo group 26.7 years^b^; *n* = 3 (5%) female	UK	2	Oral imipramine 75, *n* = 30	Placebo, *n* = 29	4 weeks	10 cm VAS, BDI
Johnson et al. [68] (crossover)	14 participants with chronic LBP; mean age 36.93 (13.05) years; *n* = 0 (0%) female	USA	2	Oral duloxetine, 30 for 1 week, titration to 60 for 2 weeks, then maintenance for 4 weeks, 30 for final week, *n* = 7 ^a^	Placebo, *n* = 7^a^	8 weeks/phase with 1-week washout	BPI
Katz et al. [69] (crossover)	54 participants with chronic LBP; mean age 50.6 (10.7) years; *n* = 21 (48%) female^c^	USA	2	Oral bupropion 150 for 3 days, 300 until end week 5, 150 until week 7, *n* = 21^a^	Placebo, *n* = 23^a^	7 weeks/phase with 2-week washout	11-point NRS, BDI, RMDQ
Konno et al. [53]	458 participants with chronic LBP; mean age 58.9 (13.4) years; *n* = 237 (52%) female	Japan; 58 sites	2	Oral duloxetine 20 first week, 40 second week, 60 weeks 3–14, *n* = 232	Placebo, *n* = 226	14 weeks	11-point NRS, RMDQ
NCT00227292 (withdrawn)	Chronic LBP	Germany	2	Oral escitalopram 10 for 1 week, 20 for 3 weeks	Placebo	4 weeks	VAS, HDRS
NCT01225068	40 participants with chronic neuropathic LBP; mean age 47.7 (10.3) years; *n* = 21 (52%) female	USA	2	Oral milnacipran 100, option to increase to 200 after 2 weeks, *n* = 20. Drug escalated in week 1 and discontinued after week 6	Placebo, *n* = 20	6 weeks	100 mm VAS
NCT03249558 (ongoing)	Chronic LBP or chronic neck pain	USA	3	Oral morphine 60 plus duloxetine, or morphine plus placebo duloxetine, or placebo morphine plus duloxetine 60	Placebo	10 weeks	VAS
NCT03364075 (crossover; terminated)	Chronic LBP	NR	3	Oral duloxetine 30 for 1 week then 60 for 1 week plus placebo, or propranolol 40 for 1 week then 60 for 1 week plus placebo, or duloxetine 30 for 1 week then 60 for 1 week plus propranolol 40 for 1 week then 60 for 1 week	Placebo	2 weeks/phase with 1-week washout	Pain index
Pheasant et al. [54] (crossover)	16 participants with chronic LBP; mean age 47.2 years^b^; *n* = 16 (75%) female	USA	2	Oral amitriptyline 50, *n* = 6^a^	Active placebo (atropine) 0.2, *n* = 10^a^	6 weeks/phase with 2-week washout	Functional evaluation rating
Schliessbach et al. [55] (crossover)	50 participants with chronic LBP; mean age 54.4 (17.3) years; *n* = 32 (64%) female	Switzerland	2	Oral imipramine 75 single dose, *n* = 50	Active placebo (tolderodine) 1.0, single dose, *n* = 50	2 h/phase with 1-week washout	11-point NRS
Schukro et al. [56] (crossover)	41 participants with chronic LBP and leg pain; mean age 57.9 years (13.4); *n* = 21 (51%) female	Austria	2	Oral duloxetine 30 to 60 first week; 60 to 120 second week; 120 for 2 weeks, *n* = 16^a^	Placebo, *n* = 18^a^	4 weeks/phase with 2-week washout	10 cm VAS, BDI, RMDQ
Skljarevski et al. [57]	236 participants with chronic LBP, mean age duloxetine groups 51.8 (14.9) years; placebo group 51.2 (13.5) years; *n* = 144 (61%) female	18 clinical sites in Brazil, France, Germany, Mexico, and Netherlands	2	Oral duloxetine 30 for 1 week, 60 for 6 weeks, non-responders increased to 120/day for remainder of study, *n* = 115	Placebo, *n* = 121	13 weeks	11-point NRS, BDI-II, RMDQ
Skljarevski et al. [58]	404 participants with chronic LBP; mean age duloxetine 20 mg group 52.9 (12.8) years, duloxetine 60 mg group 53.3 (14.7) years, duloxetine 120 mg group 54.9 (14.8) years, placebo group 54 (13.5) years; *n* = 232 (57%) female	NR	4	Oral duloxetine 20, *n* = 59, or 60, *n* = 116, or 120, *n* = 112	Placebo, *n* = 117	13 weeks	11-point NRS, BDI-II, RMDQ
Skljarevski et al. [59]	401 participants with chronic LBP, mean age duloxetine group 54.9 (13.7) years; placebo group 53.4 (14.2) years; *n* = 246 (61%) female	27 sites in Germany, Netherlands, Poland, Russia, Spain, and USA	2	Oral duloxetine 60, *n* = 198	Placebo, *n* = 203	12 weeks	11-point NRS, BDI-II, RMDQ
Treves et al. [60]	68 participants with chronic LBP, LBP with leg pain, and sciatica; mean age 45.6^b^ years; *n* = 35 (51%) female	France	3	IV clomipramine, progressive doses in the morning until maximum dose of 75 reached (3rd day) and maintained for the next 7 days with placebo in evening, *n* = 25, or IV clomipramine progressive doses in the evening until maximum dose of 75 reached (3rd day) and maintained for the next 7 days with placebo in morning, *n* = 27	IV placebo (isotonic glucose), *n* = 16	10 days	10 cm VAS
Urquhart et al. [61]	146 participants with chronic LBP; mean age amitriptyline group 53.5 (14.2) years, placebo group 56.0 (13.2) years; *n* = 56 (38.%) female	Australia	2	Oral amitriptyline 25, *n* = 72	Active placebo (benztropine mesylate) 1.0, *n* = 74	6 months	100 mm VAS, BDI, RMDQ

*NR* not reported, *LBP* low back pain, *SBPQ* Short Back Pain Questionnaire, *BDI* Beck Depression Inventory, *BDI-II* Beck Depression Inventory II, *DDS* Descriptor Differential Scale, *RMDQ* Roland Morris Disability Questionnaire, *VAS* visual analog scale, *MADRS* Montgomery Asberg Depression Rating Scale, *NRS* numerical rating scale, *BPI* Brief Pain Inventory, *HDRS* Hamilton Depression Rating Scale

^a^Number of participants randomized in the first phase of crossover trial

^b^No measure of central tendency reported

^c^Age and sex data presented for intention-to-treat sample

Eighteen trials used a parallel design, and five trials used crossover designs. Four trials were reported in trial registries. We identified a single ongoing trial, a single withdrawn trial, and a single terminated trial. Seventeen trials provided data for inclusion in the meta-analysis. These 17 trials randomized a total 2517 participants to one or more of 11 different antidepressant medicines or placebo. We did not identify any trials of antidepressant medicines compared to waiting list, usual care or no-treatment. The analyses presented below are for the effect of antidepressant medicines compared to placebo.

### Risk of bias

We assessed completed trials (*n* = 20) for overall risk of bias (Table S1 in Additional file 2); 15 were assessed as high risk, four at moderate risk, and a single trial at low risk of bias. All twenty trials reported an appropriate method of blinding. Fourteen trials reported either high dropout rates or differences in dropouts between arms. Seven trials reported that they maintained complete control over the publication of results or had no funding-related conflicts of interests.

### Assessment of publication bias

Visual inspection of funnel plots for each outcome suggested that the effects were evenly distributed around the mean (Figures S1-14 in Additional file 2). For all outcomes, visual inspection of contour-enhanced funnel plots provided no evidence of effects clustered around the threshold for statistical significance. Egger’s tests were conducted for outcomes with 10 studies; only a single study indicated statistically significant asymmetry. A single completed trial report from a trial registry (NCT01225068) was included in our analyses.

### Confidence in evidence

The GRADE assessment of confidence in the evidence for each main analysis is presented in Appendix S2 in Additional file 2 and referred to below.

## Main analysis

### Primary outcome: pain

**Fig. 2:**
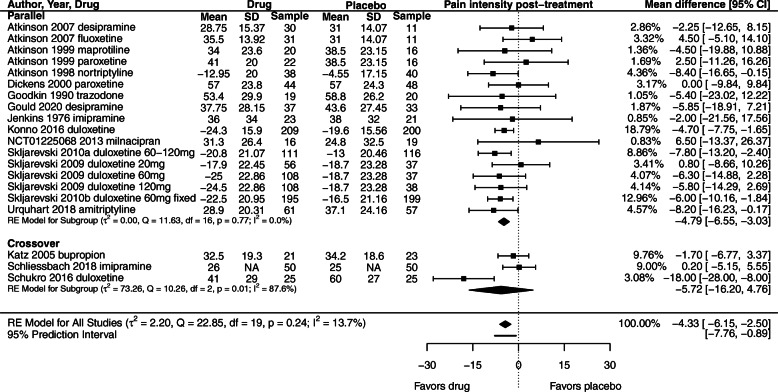
Effect of antidepressants compared to placebo on pain intensity (0–100 scale) for patients with LBP. Negative values for mean outcomes indicate change from baseline. Negative values for mean difference indicate effect favors drug compared to placebo. NA= group SD data not available; between-group summary statistics used in meta-analysis

Sixteen of the 23 included trials reported data for pain. We downgraded confidence in the evidence by two levels due to trial limitations. There is low confidence that the pooled effect of antidepressant medicines compared to placebo is − 4.33 [95% CI − 6.15 to − 2.50; Tau^2^ = 2.20] on a 0–100 point scale (Fig. 2).

### Primary outcome: acceptability

**Fig. 3:**
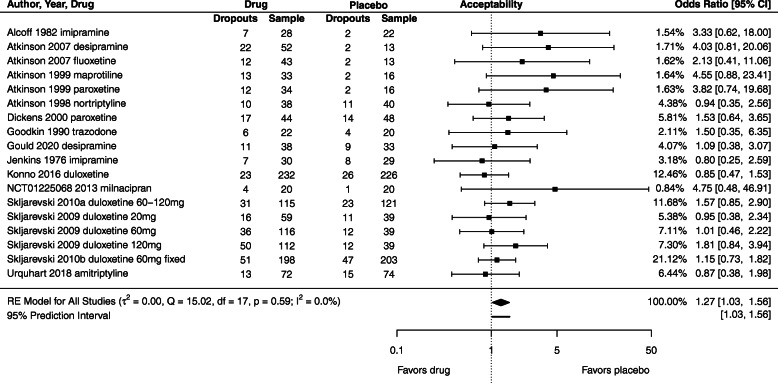
All-cause discontinuation (acceptability) of antidepressants compared to placebo for patients with LBP. Odds ratio greater than 1 indicates greater odds of discontinuation in antidepressant group (i.e., effect favors placebo)

Fourteen of the 23 included trials reported data for acceptability (all-cause discontinuation). We downgraded confidence in the evidence by two levels due to trial limitations. There is low confidence that the odds of all-cause discontinuation are higher for antidepressants than for placebo: odds ratio 1.27 [95% CI 1.03 to 1.56; Tau^2^ = 0] (Fig. 3).

### Secondary outcome: function

Six of the 23 included trials reported data for function. We downgraded confidence in the evidence by two levels due to trial limitations. There is low confidence that the pooled effect of antidepressants compared to placebo is − 3.22 [95% CI − 4.96 to − 1.48, Tau^2^ = 0] on a 0–100 point scale (Figure S15 in Additional file 2).

### Secondary outcome: symptoms of depression

Four of the 23 included trials reported data for symptoms of depression. We downgraded confidence in the evidence by two levels for trial limitations and an additional level for imprecision. There is very low confidence that the pooled effect of antidepressants compared to placebo is − 1.72 [95% CI − 3.88 to 0.44; Tau^2^ = 0] (Figure S16 in Additional file 2) on a 0–100 point scale.

### Secondary outcome: safety

Nine of the 23 included trials reported data for safety (adverse effects). We downgraded confidence in the evidence by two levels for trial limitations. There is low confidence that the odds of experiencing an adverse effect are higher for antidepressants than for placebo: odds ratio 1.58 [95% CI 1.28 to 1.93; Tau^2^ = 0] (Figure S17 in Additional file 2).

### Secondary outcome: harm

Six of the 23 included trials reported data for harm (serious adverse effects). We downgraded confidence in the evidence by two levels for trial limitations and an additional level for imprecision. There is very low confidence that the odds of experiencing a serious adverse effect are higher for antidepressants than for placebo: odds ratio 1.29 [95% CI 0.56 to 2.94; Tau^2^ = 0] (Figure S18 in Additional file 2).

### Secondary outcome: tolerability

Ten of the 23 included trials reported data for tolerability (discontinuation due to adverse effects). We downgraded confidence in the evidence by two levels for trial limitations. There is low confidence that the odds of discontinuing treatment due to an adverse effect are higher for antidepressants than for placebo: odds ratio 2.39 [95% CI 1.71 to 3.34; Tau^2^ = 0] (Figure S19 in Additional file 2).

## Other analyses

### Subgroup analyses

**Fig. 4:**
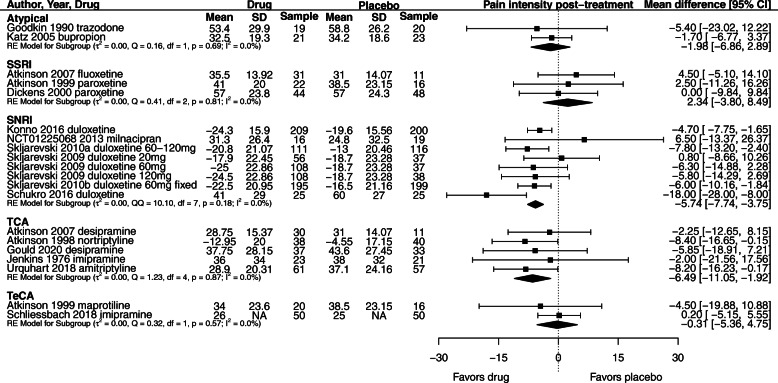
Effect of antidepressant class compared to placebo on pain intensity (0–100 scale) for patients with LBP. Negative values for mean outcomes indicate change from baseline. Negative values for mean difference indicate effect favors drug compared to placebo. NA = group SD data not available; between-group summary statistics used in meta-analysis

We conducted subgroup analyses for pain by antidepressant type and dose to provide additional clinical information (Fig. 4). There were no trials that evaluated the efficacy of HCA or MAOI antidepressants on LBP symptoms. The results for additional subgroup and sensitivity analyses are presented in Supplementary results with corresponding forest plots in Figures S20-23 in Additional file 2.

### Influence of further research on results

The extended funnel plots (Figures S24, S25 in Additional file 2) suggest the upper bound of the confidence interval for the pooled effect would cross the threshold for clinical meaningfulness if the meta-analysis included an additional hypothetical trial with approximately 400 participants per arm and an effect for pain of approximately − 30 on a 0–100 scale (antidepressants more favorable than placebo).

### Post hoc effects of duloxetine

Duloxetine is noted in the 2017 American College of Physicians guideline to have small effects on pain and function compared to placebo, for chronic LBP [11]. We repeated the main analyses on five trials that evaluated duloxetine compared to placebo. The effect of duloxetine on pain intensity post-treatment was − 5.87 [95% CI − 7.88 to − 3.86; Tau^2^ = 0] (Figure S26 in Additional file 2). The odds ratio for all-cause discontinuation of duloxetine compared to placebo was 1.17 [95% CI 0.90 to 1.52; Tau^2^ = 0] (Figure S27 in Additional file 2). The odds ratio for experiencing adverse effects of duloxetine compared to placebo was 1.50 [95% CI 1.21 to 1.85; Tau^2^ = 0] (Figure S28 in Additional file 2). The odds ratio for experiencing serious adverse effects of duloxetine compared to placebo was 1.35 [95% CI 0.56 to 3.27; Tau^2^ = 0] (Figure S29 in Additional file 2). The odds ratio for discontinuing treatment due to adverse effects of duloxetine compared to placebo was 2.53 [95% CI 1.70 to 3.77; Tau^2^ = 0] (Figure S30 in Additional file 2).

### Post hoc sensitivity analyses

The REML estimator may underestimate between-study variance for binary outcomes when events are rare [70]. We repeated the analyses for acceptability, safety, harm, and tolerability using DerSimonian-Laird, Paule and Mandel and Mantel-Haenszel methods of estimation (Table S2 in Additional file 2). A single additional post hoc sensitivity analysis is reported in Supplementary Results and Figure S31 in Additional file 2.

## Discussion

We conducted a systematic review to evaluate the effect of antidepressant medicines for patients with LBP. We included 23 trials in the systematic review and up to 17 in the meta-analyses. There is low confidence in evidence that, on average, patients with LBP treated with antidepressant medicines will experience a small improvement in pain and function and no improvement in symptoms of depression, compared to placebo. These effects are not clinically important [42, 71]. There is low confidence in evidence that patients are at increased odds of experiencing an adverse or serious adverse effect and at increased odds of stopping treatment due to an adverse effect or another reason, compared to placebo. Taken together, these data indicate treatment of LBP symptoms with antidepressants has no important benefit; is less acceptable, less safe and less tolerable; and may be harmful, compared to treatment with a placebo medicine.

A recent overview of clinical guidelines reported that 6 of 8 international guidelines recommend the use of antidepressants for chronic LBP where necessary [72]. The current American College of Physicians clinical guideline for the management of LBP [11] recommends the use of duloxetine for chronic LBP as second-line therapy where non-pharmacological therapy has been unsuccessful. This might be reconsidered in view of our findings. The analyses of duloxetine specifically showed a small effect on pain that is unlikely clinically important [73] and higher odds of adverse effects and dropout due to adverse effects compared to placebo.

Our work has a number of strengths. We adhered to a prospectively registered protocol and reported findings in line with recommendations [74]. Our searches are extensive and up to date and we included data from trial registry reports [29, 75, 76]. We also evaluated the acceptability, safety, harm, and tolerability of antidepressant medicines, in addition to effects on symptoms. This addresses limitations of the most recent review, which included 11 fewer trials and did not evaluate adverse effects [23]. The observed low heterogeneity across all outcomes, together with the improved precision of the estimates, substantiates our findings and interpretation. We determined that different methods of estimation did not influence these observations and note that similar homogeneity for binary outcomes has been reported in other large meta-analyses for antidepressant medicines [15]. We estimated parameters for a hypothetical future trial that would meaningfully impact the effect estimate for pain, to assist readers’ interpretation of the need for further trials.

We were unable to estimate effects for the long-term efficacy and acceptability of antidepressants because such data were reported in a single trial [61]. We were also unable to evaluate the effects of antidepressants in patients with acute LBP because we identified no usable data. The hypothetical future trial parameters estimated with the extended funnel plot do not consider risk of bias and are not estimable for binary outcomes.

## Conclusion

This review demonstrates that treatment of LBP symptoms with antidepressants has no important benefit; is less acceptable, less safe, and less tolerable; and may be harmful, compared to treatment with a placebo medicine. This evidence is supported by homogenous, precise effect sizes across outcomes. These findings provide Level I evidence to guide clinicians in their use of antidepressants to treat LBP.

## Supplementary Information

**Additional file 1.** PRISMA 2009 Checklist.

**Additional file 2.** Supplementary Content.

## Data Availability

The dataset used and analyzed during the current study are available from the corresponding author on reasonable request.

## Abbreviations

BDI: Beck Depression Inventory
BDI-II: Beck Depression Inventory II
BPI: Brief Pain Inventory
DDS: Descriptor Differential Scale
GRADE: Grading of Recommendations Assessment Development and Evaluation
HCA: Heterocyclic antidepressant
HDRS: Hamilton Depression Rating Scale
LBP: Low back pain
MADRS: Montgomery Asberg Depression Rating Scale
MAOI: Monoamine oxidase inhibitor
NRS: Numerical rating scale
NSAID: Non-steroidal anti-inflammatory
PRISMA: Preferred Reporting Items for Systematic Reviews and Meta-Analyses
RCT: Randomized controlled trial
REML: Restricted maximum likelihood
RMDQ: Roland Morris Disability Questionnaire
SBPQ: Short Back Pain Questionnaire
SNRI: Serotonin and norepinephrine reuptake inhibitor
SSRI: Selective serotonin reuptake inhibitor
TCA: Tricyclic antidepressant
TeCA: Tetracyclic antidepressant
VAS: Visual analog scale
