# Supplementary material for: Efficacy, acceptability, and safety of antidepressants for low back pain: a systematic review and meta-analysis
Source: Syst Rev. 2021 Feb 24;10:62. doi: 10.1186/s13643-021-01599-4 (PMC7905649; doi:10.1186/s13643-021-01599-4)
Supplement: Supplementary file 2 — Additional file 2. Supplementary Content. [file 13643_2021_1599_MOESM2_ESM.docx]

**Additional File 2 – Supplementary Content**

Ferraro et al. Efficacy, acceptability and safety of antidepressants for low back pain: a systematic review and meta-analysis

Checklist S1: PRISMA Checklist

Appendix S1: Search strategies

Table S1. Risk of bias assessment of 20 included trials

Figure S1. Funnel plot for pain intensity for trials comparing antidepressants to placebo

Figure S2. Contour-enhanced funnel plot for pain intensity for trials comparing antidepressants to placebo

Figure S3. Funnel plot for all-cause discontinuation for trials comparing antidepressants to placebo

Figure S4. Contour-enhanced funnel plot all-cause discontinuation for trials comparing antidepressants to placebo

Figure S5. Funnel plot for function for trials comparing antidepressants to placebo

Figure S6. Contour-enhanced funnel plot for function for trials comparing antidepressants to placebo

Figure S7. Funnel plot for symptoms of depression for trials comparing antidepressants to placebo

Figure S8. Contour-enhanced funnel plot for symptoms of depression for trials comparing antidepressants to placebo

Figure S9. Funnel plot for adverse effects for trials comparing antidepressants to placebo

Figure S10. Contour-enhanced funnel plot for adverse effects for trials comparing antidepressants to placebo

Figure S11. Funnel plot for serious adverse effects for trials comparing antidepressants to placebo

Figure S12. Contour-enhanced funnel plot for serious adverse effects for trials comparing antidepressants to placebo

Figure S13. Funnel plot for discontinuation due to adverse effects for trials comparing antidepressants to placebo

Figure S14. Contour-enhanced funnel plot for discontinuation due to adverse effects for trials comparing antidepressants to placebo

Appendix S2: GRADE assessment of confidence in the evidence

Figure S15. Effect of antidepressants compared to placebo on function (0-100 scale) for patients with LBP

Figure S16. Effect of antidepressants compared to placebo on symptoms of depression (0-100 scale) for patients with LBP

Figure S17. Adverse effects of antidepressants compared to placebo for patients with LBP

Figure S18. Serious adverse effects of antidepressants compared to placebo for patients with LBP

Figure S19. Discontinuation due to adverse effects of antidepressants compared to placebo for patients with LBP

Supplementary Results: Subgroup, sensitivity, and *post hoc* analyses

Figure S20. Effect of antidepressant dose compared to placebo on pain intensity (0-100 scale) for patients with LBP

Figure S21. Effect of antidepressants compared to placebo on pain intensity (0-100 scale) for patients with clearly defined non-specific LBP

Figure S22. All-cause discontinuation of antidepressants compared to placebo for patients with clearly defined non-specific LBP

Figure S23. Effect of antidepressants compared to placebo on pain intensity (0-100 scale) without studies where measures of variance were imputed

Figure S24. Extended funnel plot showing the parameters of a hypothetical trial (effect estimate, standard error) needed to shift the meta-analytic effect estimate beyond the threshold for clinical importance

Figure S25. Extended funnel plot showing the parameters of a hypothetical trial (effect estimate, sample size) needed to shift the meta-analytic effect estimate beyond the threshold for clinical importance

Figure S26. Effect of duloxetine compared to placebo on pain intensity (0-100 scale) for patients with LBP

Figure S27. All-cause discontinuation of duloxetine compared to placebo for patients with LBP

Figure S28. Adverse effects of duloxetine compared to placebo for patients with LBP

Figure S29. Serious adverse effects of duloxetine compared to placebo for patients with LBP

Figure S30. Discontinuation due to adverse effects of duloxetine compared to placebo for patients with LBP

Table S2. Comparison of Tau^2^ values with different methods of estimation for binary outcomes

Figure S31. Adverse effects of antidepressants compared to placebo for patients with LBP with the inclusion of adverse effect data for Urquhart 2018

Supplementary Reference

***Search strategy for CENTRAL – online (The Cochrane Library)
Part A: Specific search for back pain and spinal disorders***

#1 MeSH descriptor Back Pain explode all trees

#2 dorsalgia

#3 backache

#4 (lumbar next pain) or (coccyx) or (coccydynia) or (spondylosis)

#5 MeSH descriptor Coccyx

#6 MeSH descriptor Intervertebral Disc

#7 MeSH descriptor Intervertebral Disc Displacement

#8 MeSH descriptor Intervertebral Disc Degeneration

#9 MeSH descriptor Lumbar Vertebrae

#10 MeSH descriptor Sacrum

#11 MeSH descriptor Spinal Osteophytosis

#12 MeSH descriptor Spondylosis

#13 (lumbago) or (discitis) or (disc near degeneration) or (disc near prolapse) or (disc near herniation)

#14 spinal fusion

#15 facet near joints

#16 postlaminectomy

#17 failed near back

#18 slipped near (disc* or disk*)

#19 prolap* near (disc* or disk*)

#20 sciatic*

#21 back disorder*

#22 back near pain

#23 (#1 OR #2 OR #3 OR #4 OR #5 OR #6 OR #7 OR #8 OR #9 OR #10 OR #11 OR #12 OR #13 OR #14 OR #15 OR #16 OR #17 OR #18 OR #19 OR #20 OR #21 OR #22)

***Part B: Specific search for interventions of interest***

#24 agomelatine

#25 amineptine

#26 MeSH descriptor amitriptyline

#27 amitriptyline

#28 MeSH descriptor amoxapine

#29 amoxapine

#30 MeSH descriptor bupropion

#31 bupropion

#32 buproprion

#33 butriptyline

#34 MeSH descriptor citalopram

#35 citalopram

#36 escitalopram

#37 MeSH descriptor clomipramine

#38 clomipramine

#39 MeSH descriptor desipramine

#40 desipramine

#41 MeSH descriptor desvenlafaxine succinate

#42 desvenlafaxine

#43 dibenzepin

#44 dimetacrine

#45 MeSH descriptor dothiepin (MeSH for dosulepin)

#46 dothiepin

#47 dosulepin

#48 MeSH descriptor doxepin

#49 doxepin

#50 MeSH descriptor duloxetine hydrochloride

#51 duloxetine

#52 etoperidone

#53 MeSH descriptor fluoxetine

#54 fluoxetine

#55 MeSH descriptor fluvoxamine

#56 fluvoxamine

#57 gepirone
#58 MeSH descriptor imipramine

#59 imipramine

#60 MeSH descriptor isocarboxazid

#61 isocarboxazid

#62 MeSH descriptor levomilnacipran

#63 levomilnacipran

#64 MeSH descriptor maprotiline

#65 maprotiline

#66 MeSH descriptor mianserin

#67 mianserin

#68 MeSH descriptor milnacipran

#69 milnacipran

#70 MeSH descriptor mirtazapine

#71 mirtazapine

#72 MeSH descriptor moclobemide

#73 moclobemide

#74 nefazodone

#75 MeSH descriptor nortriptyline

#76 nortriptyline

#77 MeSH descriptor 5-Hydroxytryptophan

#78 hydroxytryptophan

#79 oxitriptan

#80 MeSH descriptor paroxetine

#81 paroxetine

#82 MeSH descriptor phenelzine

#83 phenelzine

#84 MeSH descriptor protriptyline

#85 protriptyline

#86 MeSH descriptor reboxetine

#87 reboxetine

#88 MeSH descriptor sertraline

#89 sertraline

#90 tianeptine

#91 MeSH descriptor tranylcypromine

#92 tranylcypromine

#93 MeSH descriptor trazodone

#94 trazodone

#95 MeSH descriptor trimipramine

#96 trimipramine

#97 MeSH descriptor tryptophan explode all trees

#98 tryptophan

#99 MeSH descriptor venlafaxine hydrochloride

#100 venlafaxine

#101 MeSH descriptor vilazodone hydrochloride

#102 vilazodone

#103 MeSH descriptor vortioxetine

#104 vortioxetine

#105 (#24 or #25 or #26 or #27 or #28 or #29 or #30 or #31 or #32 or #33 or #34 or #35 or #36 or #37 or #38 or #39 or #40 or #41 or #42 or #43 or #44 or #45 or #46 or #47 or #48 or #49 or #50 or #51 or #52 or #53 or #54 or #55 or #56 or #57 or #58 or #59 or #60 or #61 or #62 or #63 or #64 or #65 or #66 or #67 or #68 or #69 or #70 or #71 or #72 or #73 or #74 or #75 or #76 or #77 or #78 or #79 or #80 or #81 or #82 or #83 or #84 or #85 or #86 or #87 or #88 or #89 or #90 or #91 or #92 or #93 or #94 or #95 or #96 or #97 or #98 or #99 or #100 or #101 or #102 or #103 or #104) (all interventions of interest)

***Results***

#106 (#23 and #105) (all RCTs of interventions of interest in back pain)

#107 (adolescent* or teen* or youth? or puberty or childhood or children* or p?ediatri* or preschool or pre-school or nursery or kindergarten or infant? or newborn? or neonat* or prematurity or fetal or foetal)

#108 (#106 not #107) (all RCTs of interventions of interest in back pain, not children)

**Search Strategy for MEDLINE (OVID):**

***Part A: Generic search for randomized controlled trials***

1. randomized controlled trial.pt.

2. controlled clinical trial.pt.

3. comparative study.pt.

4. clinical trial.pt.

5. random*.ab.

6. placebo.ab,ti.

7. drug therapy.fs.

8. trial.ab,ti.

9. groups.ab,ti.

10. or/1-9

11. (animals not (humans and animals)).sh.

12. (adolescent* or teen* or youth? or puberty or childhood or children* or p?ediatri* or preschool or pre-school or nursery or kindergarten or infant? or newborn? or neonat* or prematurity or fetal or foetal).mp.

13. 11 or 12

14. 10 not 13

***Part B: Specific search for low back, sacrum and coccyx problems***

15. dorsalgia.ti,ab.

16. exp Back Pain/

17. backache.ti,ab.

18. ((lumb* or back) adj pain).ti,ab.

19. coccydynia.ti,ab.

20. sciatica.ti,ab.

21. spondylosis.ti,ab.

22. lumbago.ti,ab.

23. back disorder$.ti,ab

24. or/15-23

***Part C: Specific search for other spinal disorders***

25. Coccyx.sh

26. Lumbar Vertebrae.sh

27. Intervertebral disc.sh

28. discitis.ti,ab.

29. Sacrum.sh

30. Intervertebral disc degeneration.sh

31. (disc adj degenerat*).ti,ab.

32. (disc adj prolapse*).ti,ab.

33. (disc adj herniat*).ti,ab.

34. spinal fusion.sh.

35. (facet adj joint*).ti,ab.

36. Intervertebral Disc Displacement.sh.

37. postlaminectomy.ti,ab.

38. or/25-37

***Part D: Specific search for interventions of interest***

39. agomelatine.mp

40. amineptine.mp

41. exp Amitriptyline/ or amitriptyline.mp

42. amoxapine.sh or amoxapine.mp

43. bupropion.sh or bupropion.mp or buproprion.mp

44. butriptyline.mp

45. exp Citalopram/ or citalopram.mp or escitalopram.mp

46. exp Clomipramine/ or clomipramine.mp

47. desipramine.sh or desipramine.mp

48. desvenlafaxine succinate.sh or desvenlafaxine.mp

49. dibenzepin.sh or dibenzepin.mp

50. dimetacrine.mp

51. dothiepin.sh or dosulepin.mp

52. doxepin.sh or doxepin.mp

53. duloxetine hydrochloride.sh or duloxetine.mp

54. etoperidone.mp

55. fluoxetine.sh or fluoxetine.mp

56. fluvoxamine.sh or fluvoxamine.mp

57. gepirone.mp

58. imipramine.sh or imipramine.mp or imipramine oxide.mp

59. isocarboxazid.sh or isocarboxazid.mp

60. levomilnacipran.sh or levomilnacipran.mp

61. maprotiline.sh or maprotiline.mp

62. mianserin.sh or mianserin.mp

63. milnacipran.sh or milnacipran.mp

64. mirtazapine.sh or mirtazapine.mp

65. moclobemide.sh or moclobemide.mp

66. nefazodone.mp

67. nortriptyline.sh or nortriptyline.mp

68. 5-Hydroxytryptophan.sh or oxitriptan.mp

69. paroxetine.sh or paroxetine.mp

70. phenelzine.sh or phenelzine.mp

71. protriptyline.sh or protriptyline.mp

72. reboxetine.sh or reboxetine.mp

73. sertraline.sh or sertraline.mp

74. tianeptine.mp

75. tranylcypromine.sh or tranylcypromine.mp

76. trazodone.sh or trazodone.mp

77. trimipramine.sh or trimipramine.mp

78. tryptophan.sh or tryptophan.mp

79. venlafaxine hydrochloride.sh or venlafaxine.mp

80. vilazodone hydrochloride.sh or vilazodone.mp

81. vortioxetine.sh or vortioxetine.mp

82. or/39-81 (all interventions of interest)

***Results***

83. 24 or 38 (all back pain)

84. 82 and 83 (all back pain and all interventions of interest)

85. 14 and 84 (all RCTs of interventions of interest in back pain)

**Search strategy for EMBASE (Ovid):**

***Part A: Generic search for randomized controlled trials***

1 Clinical Article/

2 Clinical Study/

3 Intervention Study/

4 Longitudinal Study/

5 Prospective Study/

6 Clinical Trial/

7 Controlled Study/

8 Randomized Controlled Trial/

9 Major Clinical Study/

10 Double Blind Procedure/

11 Multicenter Study/

12 Single Blind Procedure/

13 Phase 3 Clinical Trial/

14 Phase 4 Clinical Trial/

15 crossover procedure/

16 placebo/

17 or/1-16

18 allocat$.mp.

19 assign$.mp.

20 blind$.mp.

21 (clinic$ adj25 (study or trial)).mp.

22 compar$.mp.

23 control$.mp.

24 cross?over.mp.

25 factorial$.mp.

26 follow?up.mp.

27 placebo$.mp.

28 prospectiv$.mp.

29 random$.mp.

30 ((singl$ or doubl$ or trebl$ or tripl$) adj25 (blind$ or mask$)).mp.

31 trial.mp.

32 (versus or vs).mp.

33 or/18-32

34 17 or 33

35 exp animals/ or exp invertebrate/ or animal experiment/ or animal model/ or animal tissue/ or animal cell/ or nonhuman/

36 human/ or normal human/ or human cell/

37 (adolescent* or teen* or youth? or puberty or childhood or children* or p?ediatri* or preschool or pre-school or nursery or kindergarten or infant? or newborn? or neonat* or prematurity or fetal or foetal).mp.

38 35 and 36 (animals and humans and human cells)

39 35 not 38 (animals, not animals and humans and human cells)

40 34 not 39 (RCTs not animals or animals and humans and human cells)

41 40 not 37 (RCTs not children)

***Part B: Specific search for low back, sacrum and coccyx problems***

42 dorsalgia.mp.

43 back pain.mp.

44 exp backache/

45 (lumbar adj pain).mp.

46 coccyx.mp.

47 coccydynia.mp.

48 sciatica.mp.

49 ischialgia/

50 spondylosis.mp.

51 lumbago.mp.

52 back disorder$.ti,ab.

53 or/42-52

***Part C: Specific search for other spinal disorders***

54 spine/

55 lumbar disk/

56 exp lumbar spine/

57 lumbosacral spine/

58 discitis.mp.

59 Spine Disease/

60 spinal pain/

61 spine instability/

62 spondylosis.mp

63 (disc adj degeneration).mp.

64 (disc adj prolapse).mp.

65 (disc adj herniation).mp.

66 spinal fusion.mp.

67 (facet adj joints).mp.

68 intervertebral disk.mp.

69 postlaminectomy.mp.

70 (failed adj back).mp.

71 or/54-70

***Part D: Specific search for interventions of interest***

72 agomelatine/ or agomelatine.mp

73 amineptine/ or amineptine.mp

74 amitriptyline/ or amitriptyline.mp

75 amitriptyline plus perphenazine/

76 amitriptyline plus chlordiazepoxide/

77 amoxapine/ or amoxapine.mp

78 amfebutamone/ or bupropion.mp or buproprion.mp

79 butriptyline/ or butriptyline.mp

80 citalopram/ or citalopram.mp

81 escitalopram/ or escitalopram.mp

82 clomipramine/ or clomipramine.mp

83 desipramine/ or desipramine.mp.

84 desvenlafaxine/ or desvenlafaxine.mp.

85 dibenzepin/ or dibenzepin.mp

86 dimetacrin/ or dimetacrine.mp.

87 dosulepin/ or dothiepin.mp.

88 doxepin/ or doxepin.mp.

89 duloxetine/ or duloxetine.mp.

90 duloxetine oxelate/

91 etoperidone/ or etoperidone.mp.

92 fluoxetine/ or fluoxetine.mp.

93 fluoxetine plus olanzapine/

94 fluvoaxmine/ or fluvoxamine.mp.

95 fluvoxamine maleate/

96 gepirone/ or gepirone.mp.

97 imipramine/ or imipramine.mp.

98 imipramine embonate/

99 imipraminoxide/ or imipramine oxide.mp

100 isocarboaxid/ or isocarboxazid.mp

101 milnacipran/ or milnacipran.mp.

102 levomilnacipran.mp

103 maprotiline/ or maprotiline.mp

104 mianserin/ or mianserin.mp

105 milnacipran/ or milnacipran.mp

106 mirtazapine/ or mirtazapine.mp

107 moclobemide/ or moclobemide.mp

108 nefazodone/ or nefazodone.mp

109 nortriptyline/ or nortriptyline.mp

110 5 hydroxytryptophan/ or oxitriptan.mp

111 paroxetine/ or paroxetine.mp

112 phenelzine/ or phenelzine.mp

113 protriptyline/ or protriptyline.mp

114 reboxetine/ or reboxetine.mp

115 sertraline/ or sertraline.mp

116 tianeptine/ or tianeptine.mp

117 tranylcypromine/ or tranylcypromine.mp

118 tranylcypromine derivative/

119 tranylcypromine plus trifluoperazine/

120 trazodone/ or trazodone.mp

121 trimipramine/ or trimipramine.mp

122 tryptophan/ or tryptophan.mp

123 venlafaxine/ or venlafaxine.mp

124 vilazodone.mp

125 vortioxetine/ or vortioxetine.mp

126 or/72-125 (all interventions of interest)

***Results***
127 53 or 71 (all back pain)

128 126 and 127 (all back pain and all interventions of interest)

129 41 and 128 (all RCTs of interventions of interest in back pain)

***Search strategy for CINAHL (EBSCOhost)***

***Part A: Generic search for randomized controlled trials***

S28 S26 NOT S27

S27 (MH "Animals")

S26 S7 or S12 or S19 or S25

S25 S20 or S21 or S22 or S23 or S24

S24 volunteer*

S23 prospectiv*

S22 control*

S21 followup stud*

S20 follow-up stud*

S19 S13 or S14 or S15 or S16 or S17 or S18

S18 (MH "Prospective Studies+")

S17 (MH "Evaluation Research+")

S16 (MH "Comparative Studies")

S15 latin square

S14 (MH "Crossover Design")

S13 (MH "Random Sample")

S12 S8 or S9 or S10 or S11

S11 random*

S10 placebo*

S9 (MH "Placebos")

S8 (MH "Placebo Effect")

S7 S1 or S2 or S3 or S4 or S5 or S6

S6 triple-blind

S5 single-blind

S4 double-blind

S3 clinical W3 trial

S2 "randomi?ed controlled trial*"

S1 (MH "Clinical Trials+")

***Part B: Specific search for low back, sacrum and coccyx problems***

S45 S34 or S44

S44 S35 or S36 or S37 or S38 or S39 or S40 or S41 or S42 or S43

S43 "lumbago"

S42 lumbar N2 vertebra

S41 (MH "Lumbar Vertebrae")

S40 "coccydynia" OR "back disorder*"

S39 "coccyx"

S38 "sciatica"

S37 (MH "Sciatica")

S36 (MH "Coccyx")

S35 (MH “Sacrum”)

S34 S29 or S30 or S31 or S32 or S33

S33 lumbar N5 pain

S32 lumbar W1 pain

S31 "backache"

S30 (MH "Back Pain+")

S29 "dorsalgia"

***Part C: Specific search for other spinal disorders***

S55 S46 or S47 or S48 or S49 or S50 or S51 or S52 or S53 or S54

S54 failed W1 back

S53 (MH "Laminectomy")

S52 facet W1 joint

S51 (MH "Spinal Fusion")

S50 disc W5 herniation

S49 disc W5 prolapse

S48 disc W5 degeneration

S47 (MH "Intervertebral Disk Displacement")

S46 (MH "Intervertebral Disk")

***Part D: Specific search for interventions of interest***

S103 S56 OR S57 OR S58 OR S59 OR S60 OR S61 OR S62 OR S63 OR S64 OR S65 OR S66 OR S67 OR S68 OR S69 OR S70 OR S71 OR S72 OR S73 OR S74 OR S75 OR S76 OR S77 OR S78 OR S79 OR S80 OR S81 OR S82 OR S83 OR S84 OR S85 OR S86 OR S87 OR S88 OR S89 OR S90 OR S91 OR S92 OR S93 OR S94 OR S95 OR S96 OR S97 OR S98 OR S99 OR S100 OR S101 OR S102

S103 (all interventions of interest)

S102 vortioxetine

S101 vilazodone

S100 (MH "Venlafaxine+") OR venlafaxine

S99 tryptophan

S98 trimipramine

S97 (MH "Trazodone")

S96 tranylcypromine

S95 tianeptine

S94 sertraline

S93 reboxetine

S92 protriptyline

S91 phenelzine

S90 paroxetine

S89 5-hydroxytryptophan

S88 oxitriptan

S87 (MH "Nortriptyline")

S86 nefazodone

S85 moclobemide

S84 mirtazapine

S83 milnacipran

S82 mianserin

S81 maprotiline

S80 levomilnacipran

S79 isocarboxazid

S78 (MH "Imipramine") OR "imipramine"

S77 gepirone

S76 (MH "Fluvoxamine Maleate") OR "fluvoxamine"

S75 (MH "Fluoxetine+") OR "fluoxetine"

S74 etoperidone

S73 (MH "Duloxetine Hydrochloride") OR "duloxetine"

S72 (MH "Doxepin") OR doxepin

S71 dosulepin

S70 dothiepin

S69 dimetacrine

S68 dibenzepin

S67 (MH "Desvenlafaxine Succinate") OR desvenlafaxine

S66 (MH "Desipramine") OR desipramine

S65 (MH "Clomipramine") OR clomipramine

S64 escitalopram

S63 (MH "Citalopram") OR citalopram

S62 butriptyline

S61 buproprion

S60 (MH "Bupropion") OR bupropion

S59 (MH "Amoxapine") OR amoxapine

S58 (MH "Amitriptyline") OR amitriptyline

S57 amineptine

S56 agomelatine

***Results***

S107 S106 (exclude MEDLINE records)

S106 S28 and S105 (all RCTs of interventions of interest in back pain)

S105 S103 and S104 (all back pain and all interventions of interest)

S104 S45 or S55 (all back pain)

**Search strategy for trial registries**

**ClinicalTrials.gov**

*Study Type:* Interventional Studies

*Study Results:* All studies

*Recruitment:* All studies

*Age:* Adult and Senior

*Gender:* All studies

*Conditions:* ‘back pain’

*Interventions:* all

**EU Clinical Trials Register**

*Search term:* back pain AND ‘intervention’

*Country:* -

*Age Range:* Adult & Elderly

*Trial Status:* -

*Trial Phase:* -

*Gender:* Both

*Date Range:* -

*Results Status:* -

**WHO ICTRP**

*Title:* -

*Condition:* ‘back pain’

*Intervention:* ALL

*Recruitment status:* ALL

*Phases are:* ALL

**Interventions of interest**

1. agomelatine
2. amineptine
3. amitriptyline
4. amoxapine
5. bupropion
6. butriptyline
7. citalopram
8. clomipramine
9. desipramine
10. desvenlafaxine
11. dibenzepin
12. dimetacrine
13. dosulepin
14. doxepin
15. duloxetine
16. escitalopram
17. etoperidone
18. fluoxetine
19. fluvoxamine
20. gepirone
21. imipramine
22. imipramine oxide
23. isocarboxazid
24. levomilnacipran
25. maprotiline
26. mianserin
27. milnacipran
28. mirtazapine
29. moclobemide
30. nefazodone
31. nortriptyline
32. oxitriptan
33. paroxetine
34. phenelzine
35. protriptyline
36. reboxetine
37. sertraline
38. tianeptine
39. tranylcypromine
40. trazodone
41. trimipramine
42. tryptophan
43. venlafaxine
44. vilazodone
45. vortioxetine

**Table S1. Risk of bias assessment of 20 included trials**

|  | | Random sequence generation | Allocation concealment | Blinding (Patients) | Blinding (Care-providers) | Blinding (Outcome assessors) | Drop Outs | Intention-to-treat analysis? | Selective outcome reporting | Similarity at baseline | Co-interventions | Compliance | Timing of assessment | Other bias | Overall rating |
| --- | --- | --- | --- | --- | --- | --- | --- | --- | --- | --- | --- | --- | --- | --- | --- |
| **Alcoff** | **1982** | Unclear | Unclear | Low | Low | Low | High | High | Unclear | Low | Unclear | Unclear | Low | Unclear | High |
| **Atkinson** | **2007** | Low | Low | Low | Low | Low | High | Low | Unclear | Unclear | Unclear | Unclear | Low | Low | High |
| **Atkinson** | **1999** | Low | Low | Low | Low | Low | High | Unclear | Unclear | Unclear | Low | Unclear | Low | Low | High |
| **Atkinson** | **1998** | Low | Low | Low | Low | Low | High | Low | Unclear | Low | Low | Unclear | Low | Low | High |
| **Dickens** | **2000** | Low | Low | Low | Low | Unclear | High | Low | Unclear | Low | Unclear | Unclear | Low | High | High |
| **Goodkin** | **1990** | Unclear | Unclear | Low | Low | Low | High | Unclear | Unclear | Low | Low | High | Unclear | Low | High |
| **Gould** | **2020** | Low | Low | Low | Low | Low | High | Low | Low | Unclear | Unclear | Low | Low | Low | Moderate |
| **Jenkins** | **1976** | Unclear | Unclear | Low | Low | Unclear | High | High | Unclear | Unclear | High | Unclear | Low | High | High |
| **Johnson** | **2011** | Unclear | Unclear | Low | Low | Low | Low | Unclear | Unclear | Unclear | Unclear | Unclear | Unclear | Unclear | Moderate |
| **Katz** | **2005** | Low | Unclear | Low | Low | Low | Low | High | Unclear | Unclear | Unclear | Unclear | Low | High | High |
| **Konno** | **2016** | Low | Low | Low | Low | Low | Low | Unclear | Low | Low | Low | Unclear | Low | High | High |
| **NCT01225068** | **2014** | Unclear | Unclear | Low | Low | Low | Low | High | Low | Unclear | Unclear | Unclear | Low | Low | High |
| **Pheasant** | **1983** | Unclear | Unclear | Low | Low | Low | High | Unclear | Unclear | Unclear | Low | Unclear | Low | Unclear | High |
| **Schliessbach** | **2018** | Low | Low | Low | Low | Low | Unclear | Unclear | Low | Unclear | Low | Low | Low | Unclear | Moderate |
| **Schukro** | **2016** | Low | Low | Low | Low | Low | High | Low | High | Unclear | Low | Unclear | Low | Low | High |
| **Skljarevski** | **2010a** | Unclear | Unclear | Low | Low | Low | High | Low | Low | Low | Low | Unclear | Low | High | High |
| **Skljarevski** | **2009** | Low | Unclear | Low | Low | Low | High | Low | High | Low | Low | Unclear | Low | High | High |
| **Skljarevski** | **2010b** | Unclear | Unclear | Low | Low | Low | High | Low | Low | Low | High | Low | Low | High | High |
| **Treves** | **1991** | Unclear | Unclear | Low | Unclear | Unclear | Unclear | Unclear | Unclear | Unclear | Unclear | Unclear | Low | Unclear | Moderate |
| **Urquhart** | **2018** | Low | Low | Low | Low | Low | Low | Low | Low | Low | Unclear | Low | Low | Low | Low |

**Figure S1. Funnel plot for pain intensity for trials comparing antidepressants to placebo**

Egger’s test for funnel plot asymmetry: z = 0.59, p = 0.55

**Figure S2. Contour-enhanced funnel plot for pain intensity for trials comparing antidepressants to placebo**

**Figure S3. Funnel plot for all-cause discontinuation for trials comparing antidepressants to placebo**

Egger’s test for funnel plot asymmetry: z = 2.31, p = 0.02

**Figure S4. Contour-enhanced funnel plot all-cause discontinuation for trials comparing antidepressants to placebo**

**Figure S5. Funnel plot for function for trials comparing antidepressants to placebo**

**Figure S6. Contour-enhanced funnel plot for function for trials comparing antidepressants to placebo**

**Figure S7. Funnel plot for symptoms of depression for trials comparing antidepressants to placebo**

**
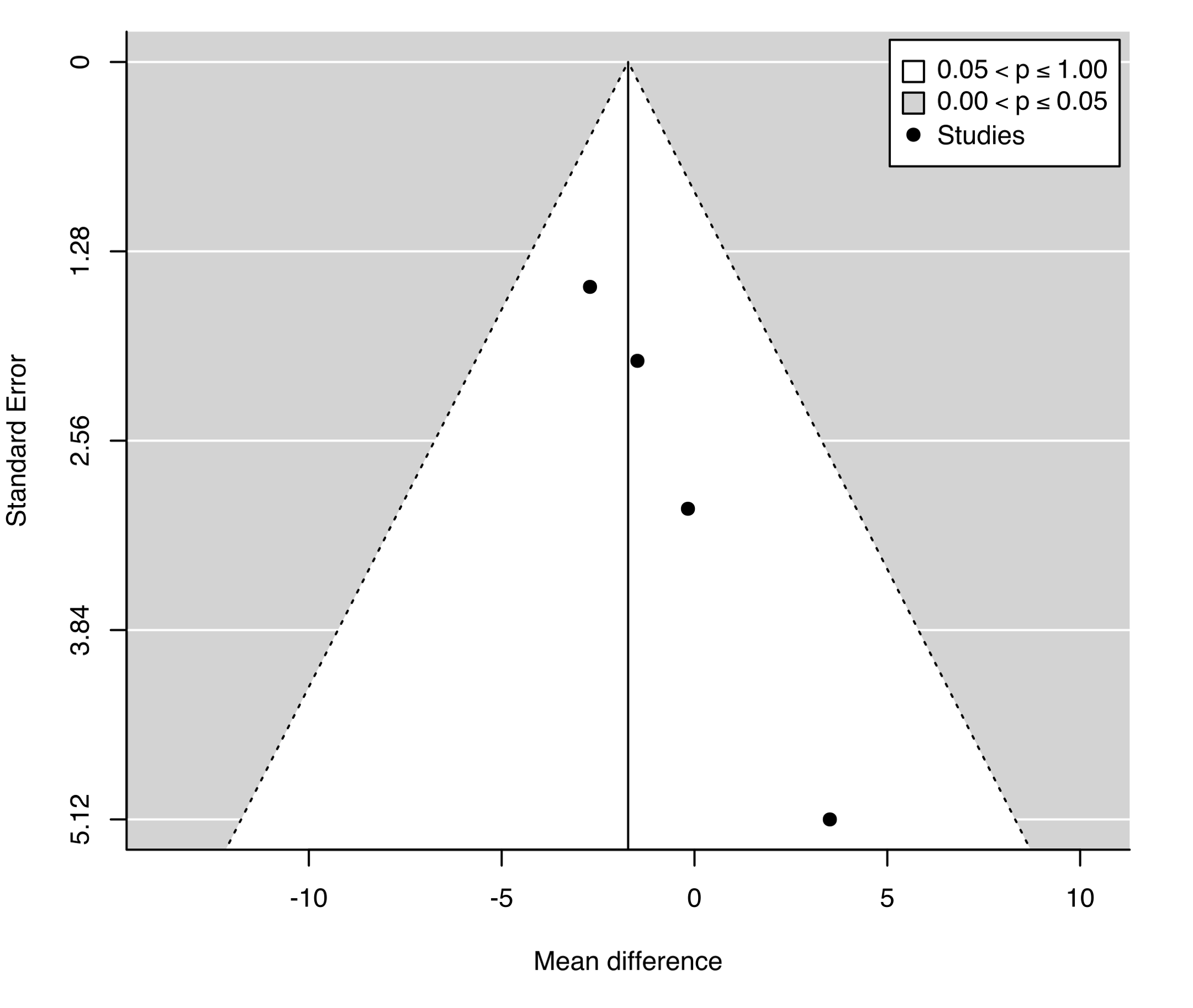
**

**Figure S8. Contour-enhanced funnel plot for symptoms of depression for trials comparing antidepressants to placebo**

**
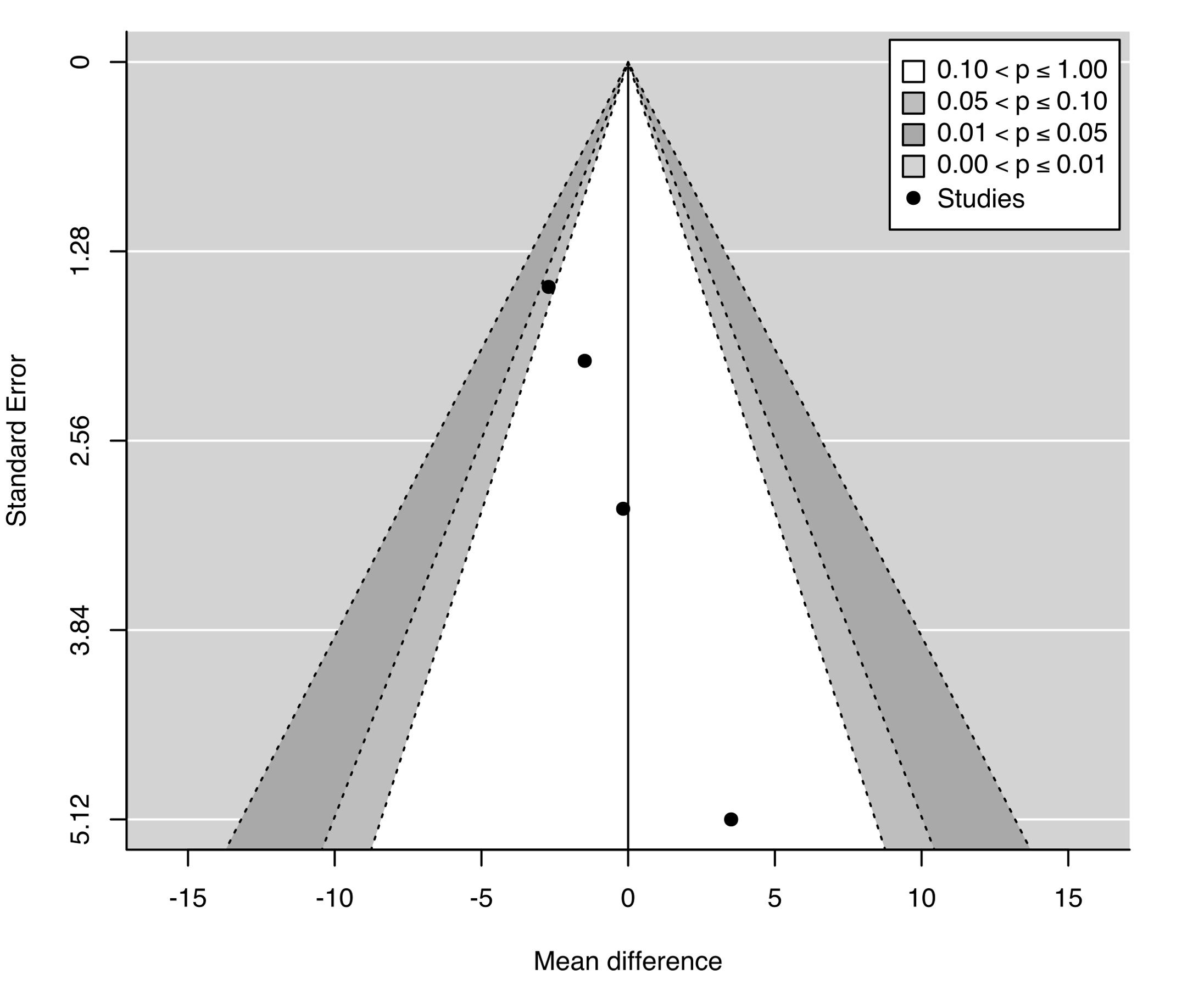
Figure S9. Funnel plot for adverse effects for trials comparing antidepressants to placebo**

Egger’s test for funnel plot asymmetry: z = 0.39, p = 0.69

**Figure S10. Contour-enhanced funnel plot for adverse effects for trials comparing antidepressants to placebo**

**Figure S11. Funnel plot for serious adverse effects for trials comparing antidepressants to placebo**

**Figure S12. Contour-enhanced funnel plot for serious adverse effects for trials comparing antidepressants to placebo**

**Figure S13. Funnel plot for discontinuation due to adverse effects for trials comparing antidepressants to placebo**

Egger’s test for funnel plot asymmetry: z = - 0.46, p = 0.63

**Figure S14. Contour-enhanced funnel plot for discontinuation due to adverse effects for trials comparing antidepressants to placebo**

**Appendix S2: GRADE assessment of confidence in the evidence**

**All Antidepressants**

Primary outcome- Pain

Risk of bias: downgrade by 2 levels, 92% of patients in trials with high ROB

Inconsistency: Tau^2^ = 2.20; I^2^ = 13.7%. Not downgraded

Indirectness: N/A

Imprecision: CIs do not span null or threshold for clinical importance

Publication bias: No sign of asymmetry on funnel plot

Quality of evidence: Low

Primary outcome- Acceptability

Risk of bias: downgrade by 2 levels, 94% of patients in trials with high ROB

Inconsistency: Tau^2^ = 0; I^2^ = 0%. Not downgraded

Indirectness: N/A

Imprecision: CIs do not span null

Publication bias: No sign of asymmetry on funnel plot

Quality of evidence: Low

Secondary outcome- Disability

Risk of bias: downgrade by 2 levels, 91% of patients in trials with high ROB

Inconsistency: Tau^2^ = 0; I^2^ = 0%. Not downgraded

Indirectness: N/A

Imprecision: CIs do not span null or threshold for clinical importance

Publication bias: No sign of asymmetry on funnel plot

Quality of evidence: Low

Secondary outcome- Depressive symptoms

Risk of bias: downgrade by 2 levels, 64% of patients in trials with high ROB

Inconsistency: Tau^2^ = 0; I^2^ = 0%. Not downgraded

Indirectness: N/A

Imprecision: CIs span null

Publication bias: No sign of asymmetry on funnel plot

Quality of evidence: Very low

Secondary outcome- Safety

Risk of bias: downgrade by 2 levels, 100% of patients in trials with high ROB

Inconsistency: Tau^2^ = 0; I^2^ = 0%. Not downgraded

Indirectness: N/A

Imprecision: CIs do not span null

Publication bias: No sign of asymmetry on funnel plot

Quality of evidence: Low

Secondary outcome- Harm

Risk of bias: downgrade by 2 levels, 100% of patients in trials with high ROB

Inconsistency: Tau^2^ = 0; I^2^ = 0%. Not downgraded

Indirectness: N/A

Imprecision: CIs span null

Publication bias: No sign of asymmetry on funnel plot

Quality of evidence: Very low

Secondary outcome- Tolerability

Risk of bias: downgrade by 2 levels, 92% of patients in trials with high ROB

Inconsistency: Tau^2^ = 0; I^2^ = 0%. Not downgraded

Indirectness: N/A

Imprecision: CIs do not span null

Publication bias: No sign of asymmetry on funnel plot

Quality of evidence: Low

**Figure S15. Effect of antidepressants compared to placebo on function (0-100 scale) for patients with LBP**

Negative values for mean outcomes indicate change from baseline. Negative values for mean difference indicate effect favors drug compared to placebo. NA = group SD data not available; between-group summary statistics used in meta-analysis

**Figure S16. Effect of antidepressants compared to placebo on symptoms of depression (0-100 scale) for patients with LBP**


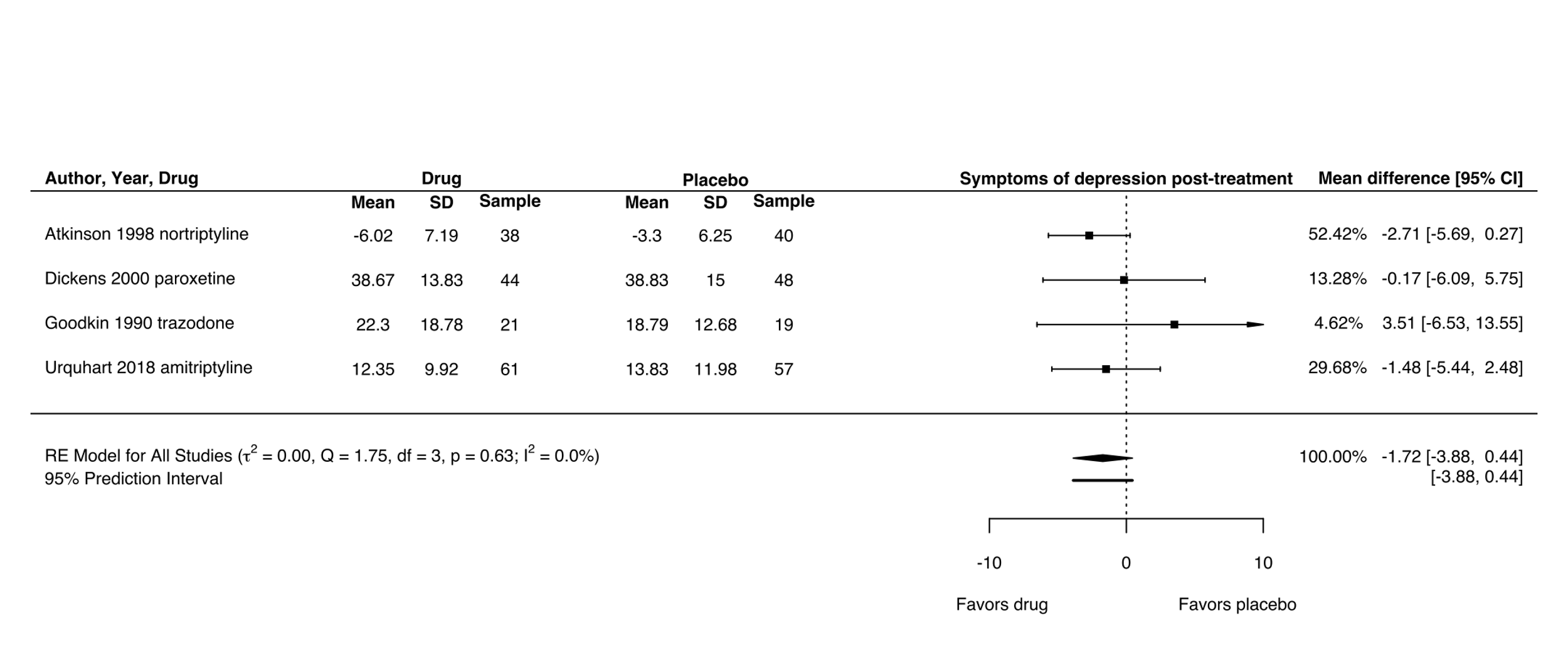


Negative values for mean outcomes indicate change from baseline. Negative values for mean difference indicate effect favors drug compared to placebo

**Figure S17. Adverse effects of antidepressants compared to placebo for patients with LBP**

Odds ratio greater than 1 indicates greater odds of discontinuation in antidepressant group (i.e. effect favors placebo)

**Figure S18. Serious adverse effects of antidepressants compared to placebo for patients with LBP**

Odds ratio greater than 1 indicates greater odds of discontinuation in antidepressant group (i.e. effect favors placebo)

**Figure S19. Discontinuation due to adverse effects of antidepressants compared to placebo for patients with LBP**

Odds ratio greater than 1 indicates greater odds of discontinuation in antidepressant group (i.e. effect favors placebo)

**Supplementary results**

**Subgroup analysis: effects of antidepressant dose on pain intensity**

The effect of antidepressants administered at standard dose compared to placebo was -4.22 [95% CI -6.08 to -2.36; Tau^2^ = 0.23]. The effect of antidepressants administered at below standard dose compared to placebo was -8.36, [95% CI -18.65 to 1.93; Tau^2^ = 60.89]. The effect of antidepressants administered at above standard dose compared to placebo was -4.84, [95% CI -9.86 to 0.19; Tau^2^ = 0] (Figure S20).

**Sensitivity analysis: studies where the definition of non-specific LBP is not clear (pain intensity)**

The exclusion of 2 studies with an unclear definition of non-specific LBP (Schukro 2016, NCT01225068) reduced the effect size for pain by 4.84% to - 4.12 [95% CI - 5.71 to - 2.52]. Tau^2^ reduced from 3.49 to 0.00 (Figure S21).

**Sensitivity analysis: studies where the definition of non-specific LBP is not clear (acceptability)**

The exclusion of 2 studies with an unclear definition of non-specific LBP (Alcoff 1982, NCT01225068) reduced the odds of all-cause discontinuation (acceptability) by 3.15% to 1.23 [95% CI 1.00 to -1.52]. Tau^2^ remained at 0 (Figure S22).

**Sensitivity analysis: studies where the measures of variance were imputed (pain intensity)**

The exclusion of studies where measures of variance were imputed (Atkinson 2007 desipramine arm and all crossover trials) increased the effect size for pain by 11.09% to -4.87 [95% CI -6.65 to -3.08]. Tau^2^ remained at 2.20 (Figure S23).

**Post hoc sensitivity analysis: inclusion of adverse effect data for Urquhart 2018**

A single study^1^ reported combined data for adverse effects (safety) and serious adverse effects (harm). We repeated the analysis for the safety outcome with the combined data included in the analysis. The inclusion of this data reduced the odds ratio by 5.70% to 1.49 [95% CI 1.22 to 1.81]. Tau^2^ remained at 0 (Figure S31).

**Figure S20. Effect of antidepressant dose compared to placebo on pain intensity (0-100 scale) for patients with LBP**

Negative values for mean outcomes indicate change from baseline. Negative values for mean difference indicate effect favors drug compared to placebo. NA = group SD data not available; between-group summary statistics used in meta-analysis

**Figure S21. Effect of antidepressants compared to placebo on pain intensity (0-100 scale) for patients with clearly defined non-specific LBP**

Negative values for mean outcomes indicate change from baseline. Negative values for mean difference indicate effect favors drug compared to placebo. NA = group SD data not available; between-group summary statistics used in meta-analysis

**Figure S22. All-cause discontinuation of antidepressants compared to placebo for patients with clearly defined non-specific LBP**

Odds ratio greater than 1 indicates greater odds of discontinuation in antidepressant group (i.e. effect favors placebo)

**Figure S23. Effect of antidepressants compared to placebo on pain intensity (0-100 scale) without studies where measures of variance were imputed**

Negative values for mean outcomes indicate change from baseline. Negative values for mean difference indicate effect favors drug compared to placebo

**Figure S24. Extended funnel plot showing the parameters of a hypothetical trial (effect, standard error) needed to shift the meta-analytic effect estimate beyond the threshold for clinical importance**

*
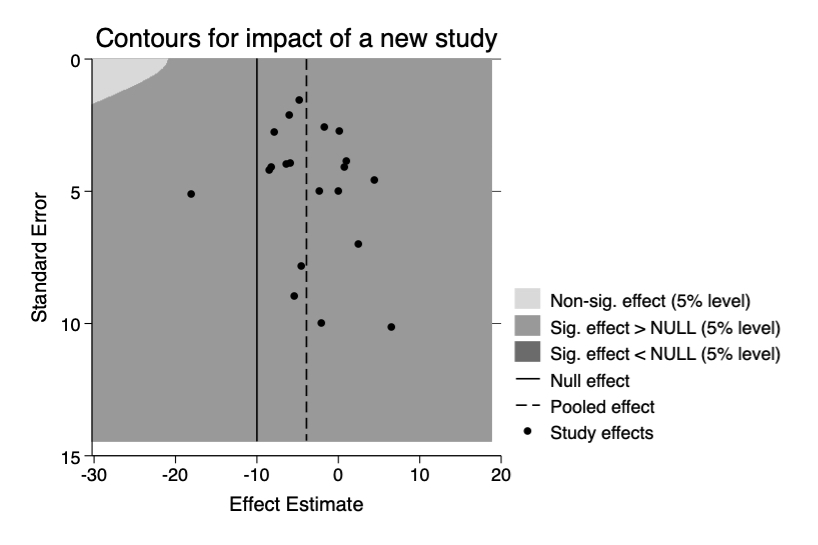
*

The unbroken line indicates the smallest worthwhile effect (10 on a 100 point-scale)

**Figure S25. Extended funnel plot showing the parameters of a hypothetical trial (effect, sample size) needed to shift the meta-analytic effect estimate beyond the threshold for clinical importance**

**
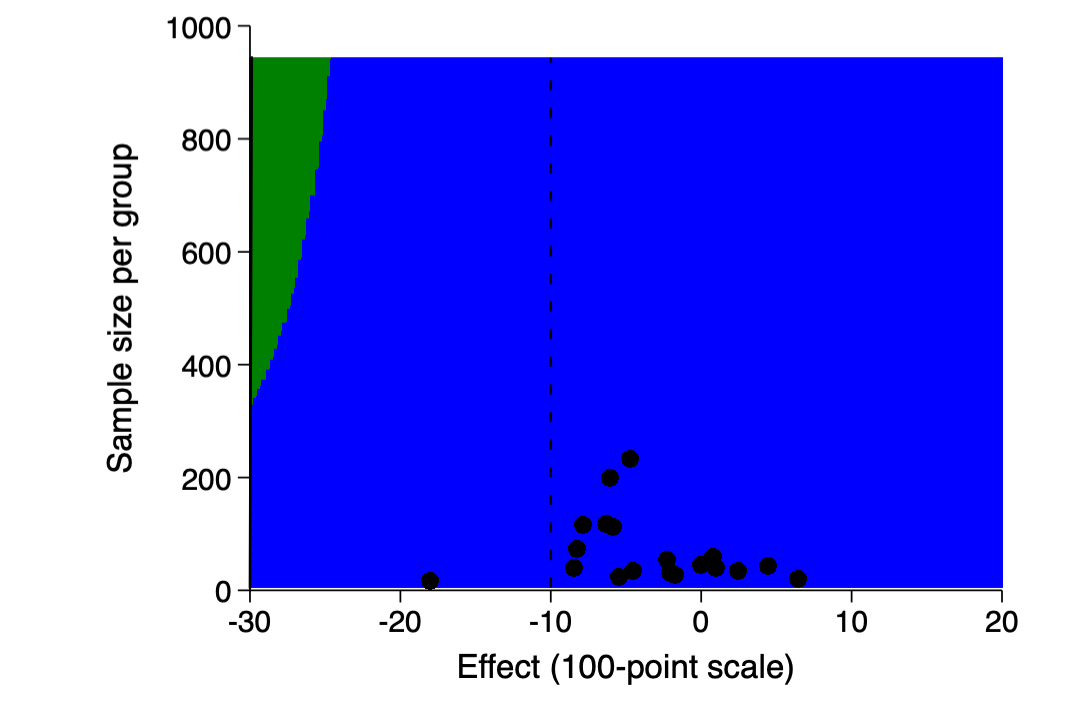
**

The broken line indicates the smallest worthwhile effect (10 on a 100 point-scale)

**Figure S26. Effect of duloxetine compared to placebo on pain intensity (0-100 scale) for patients with LBP**


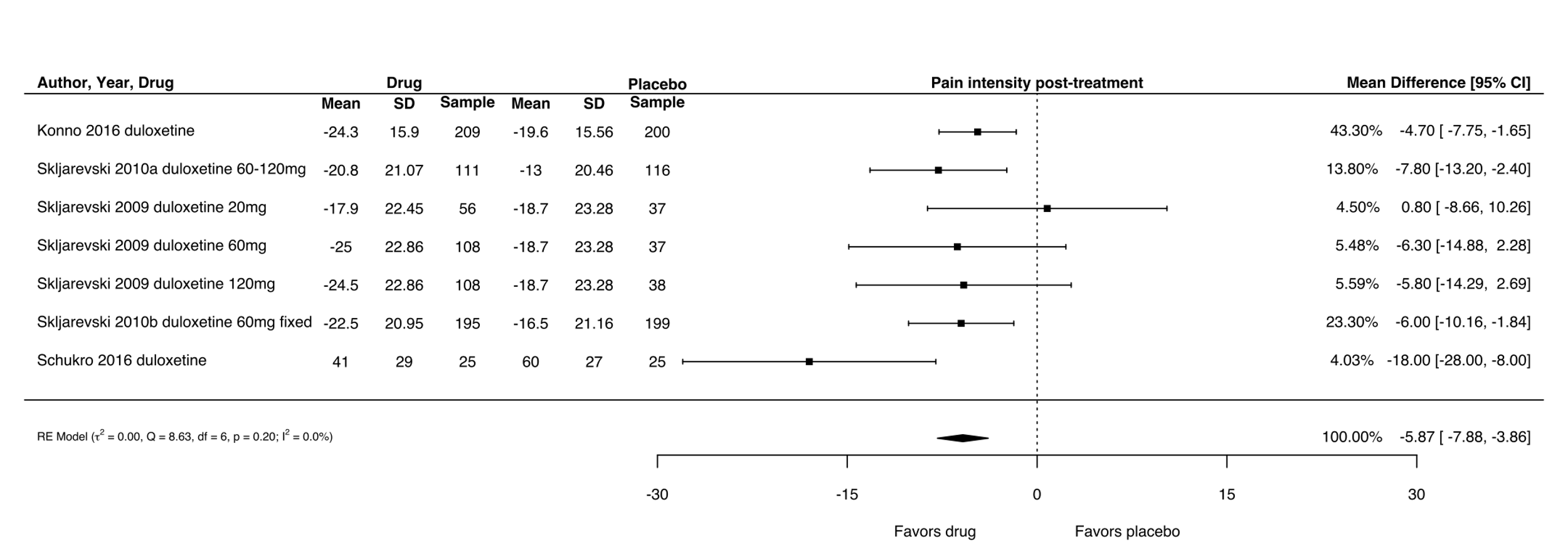


Negative values for mean outcomes indicate change from baseline. Negative values for mean difference indicate effect favors drug compared to placebo

**Figure S27. All-cause discontinuation of duloxetine compared to placebo for patients with LBP**


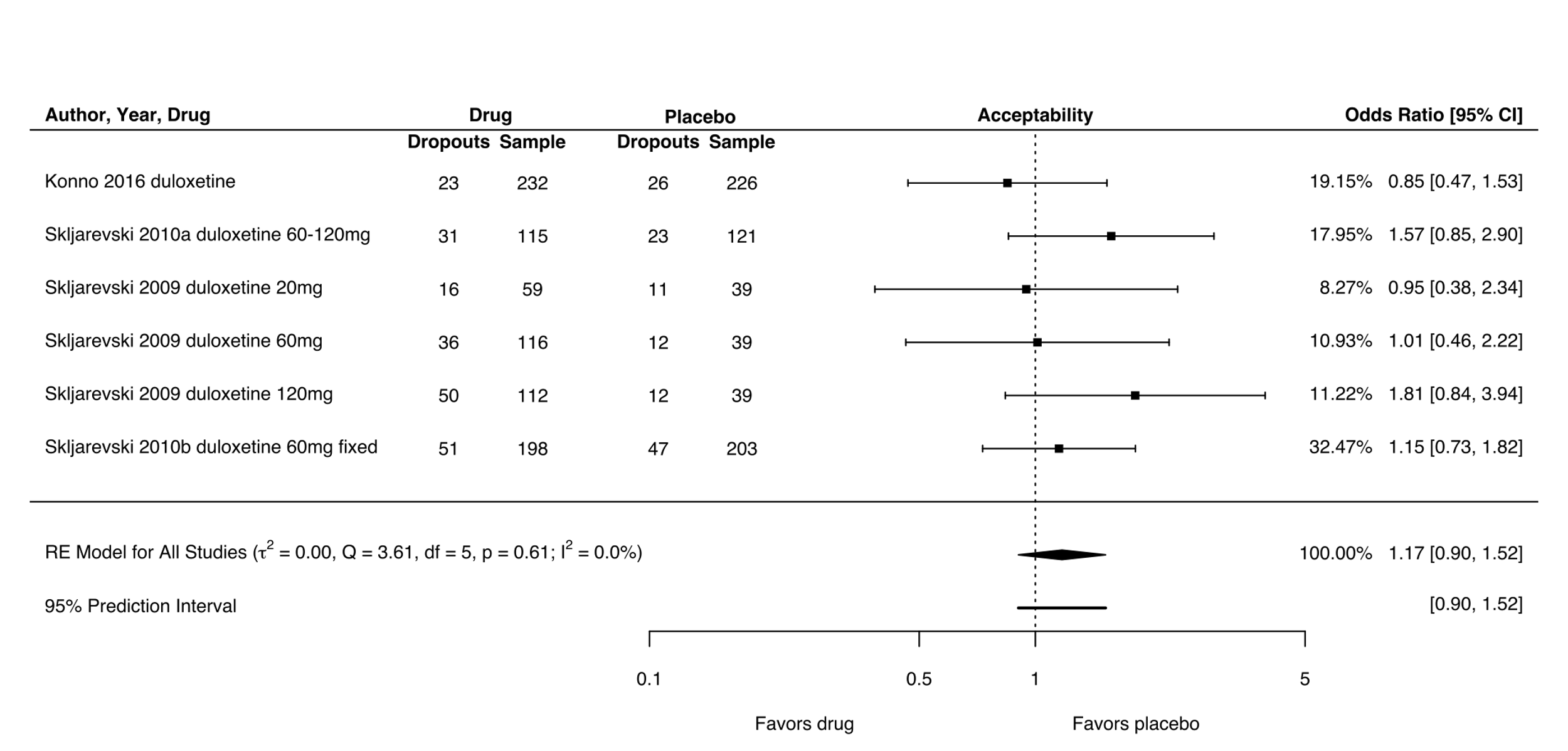


Odds ratio greater than 1 indicates greater odds of discontinuation in antidepressant group (i.e. effect favors placebo)

**Figure S28. Adverse effects of duloxetine compared to placebo for patients with LBP**


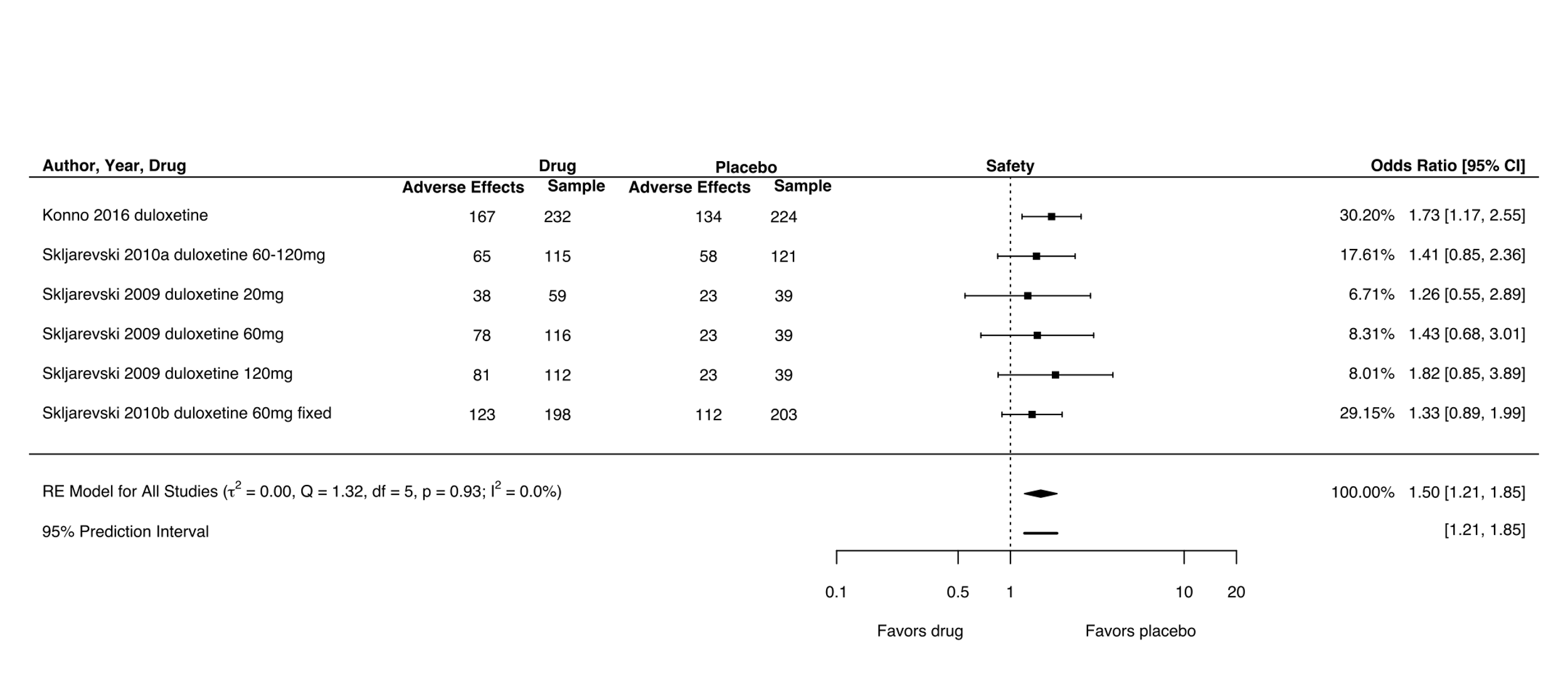


Odds ratio greater than 1 indicates greater odds of discontinuation in antidepressant group (i.e. effect favors placebo)

**Figure S29. Serious adverse effects of duloxetine compared to placebo for patients with LBP**


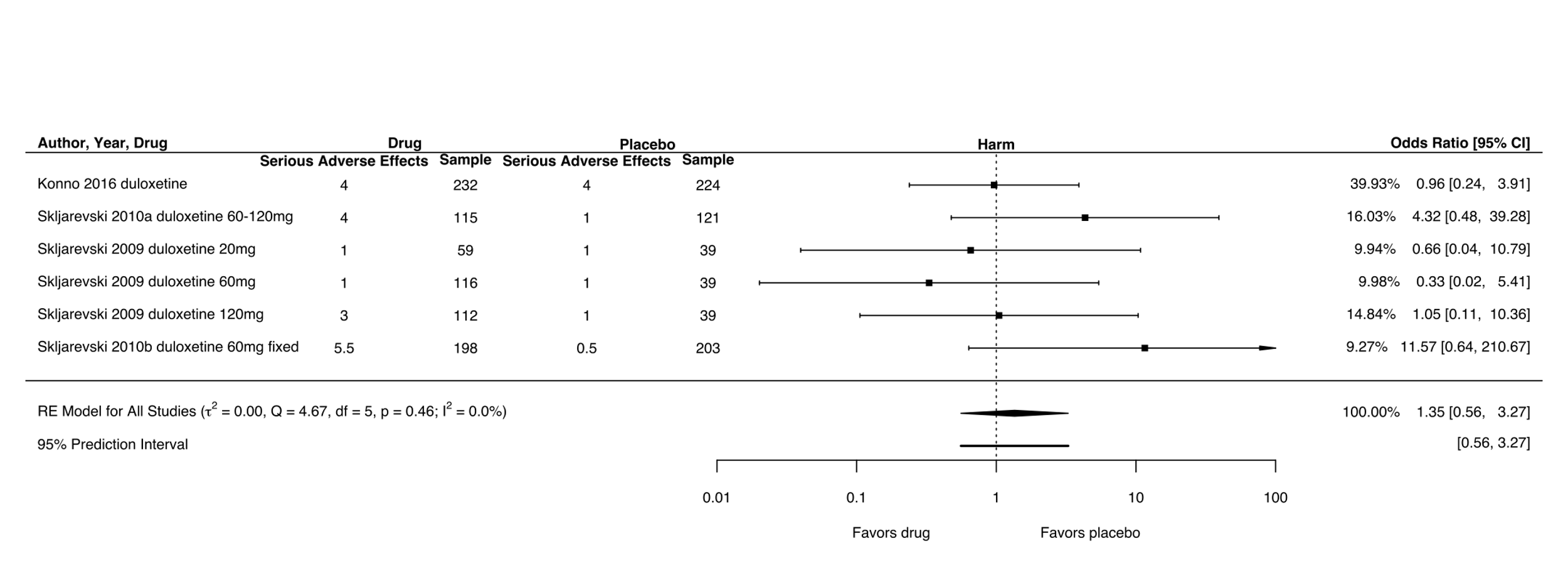


Odds ratio greater than 1 indicates greater odds of discontinuation in antidepressant group (i.e. effect favors placebo)

**Figure S30. Discontinuation due to adverse effects of duloxetine compared to placebo for patients with LBP**


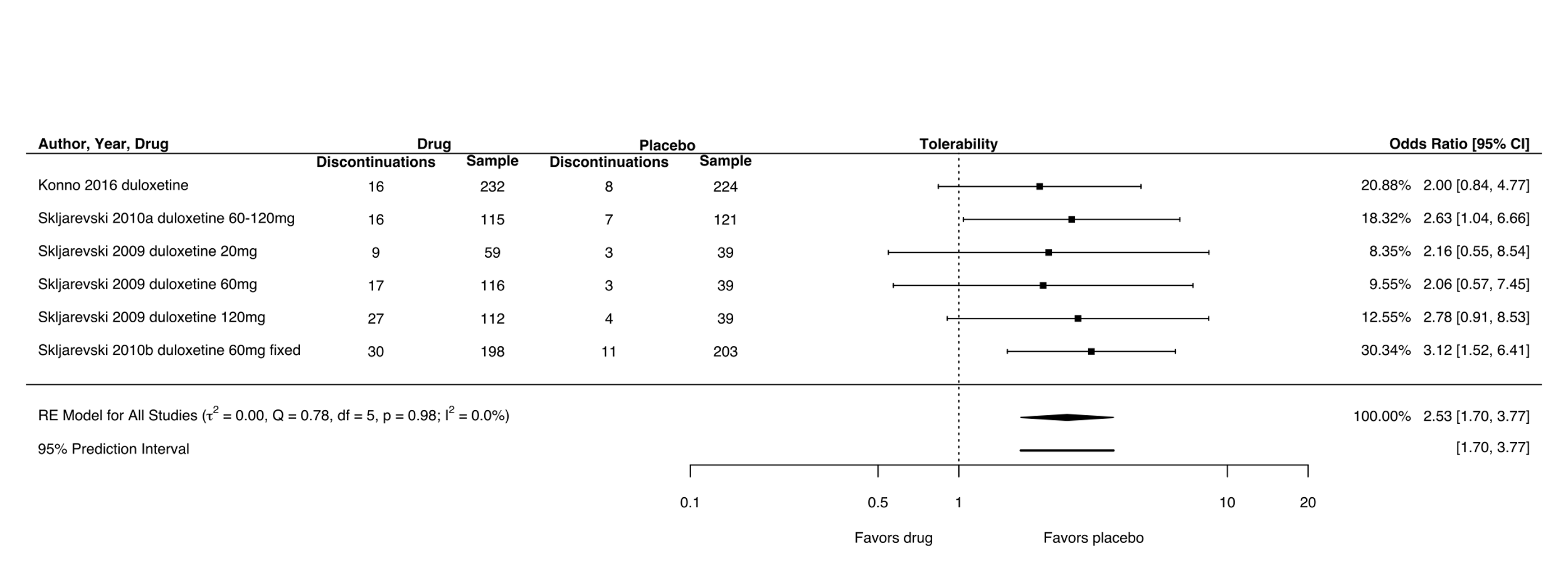


Odds ratio greater than 1 indicates greater odds of discontinuation in antidepressant group (i.e. effect favors placebo)

**Table S2. Comparison of Tau^2^ values with different methods of estimation for binary outcomes**

| **Outcome** | **REML** | **DerSimonian-Laird** | **Paule & Mandel** | **Mantel-Haenszel** |
| --- | --- | --- | --- | --- |
| Acceptability | 0 | 0 | 0 | 0 |
| Safety | 0 | 0.02 | 0.04 | 0 |
| Harm | 0 | 0 | 0 | 0 |
| Tolerability | 0 | 0.02 | 0.02 | 0 |

**Figure S31. Adverse effects of antidepressants compared to placebo for patients with LBP with the inclusion of adverse effect data for Urquhart 2018**^1^

Odds ratio greater than 1 indicates greater odds of discontinuation in antidepressant group (i.e. effect favors placebo)

**Supplementary References**

1. Urquhart DM, Wluka AE, van Tulder M, et al. Efficacy of low-dose amitriptyline for chronic low back pain. *JAMA Intern Med*. 2018;178(11):1474. doi:10.1001/jamainternmed.2018.4222
